# How Effective Are the Canine Visceral Leishmaniasis Vaccines Currently Being Tested in Dogs? A Systematic Review and Meta‐Analysis

**DOI:** 10.1111/pim.70006

**Published:** 2025-03-03

**Authors:** Josiane Aparecida Martiniano de Pádua, Tuane Ferreira Melo, Rafaella Silva Andrade, Marina Martins de Oliveira, Ana Laura Grossi de Oliveira, Andressa Mariana Saldanha‐Elias, Ricardo Toshio Fujiwara, Elaine Maria Seles Dorneles, Ana Paula Peconick, Kelly Moura Keller

**Affiliations:** ^1^ Departamento de Medicina Veterinária Preventiva, Escola de Veterinária Universidade Federal de Minas Gerais—UFMG Belo Horizonte Brazil; ^2^ Departamento de Medicina Veterinária, Faculdade de Zootecnia e Medicina Veterinária Universidade Federal de Lavras—UFLA Lavras Brazil; ^3^ Programa de Pós‐Graduação em Ciências da Saúde: Doenças Infecciosas e Medicina Tropical, Faculdade de Medicina Universidade Federal de Minas Gerais—UFMG Belo Horizonte Brazil; ^4^ Departamento de Parasitologia, Instituto de Ciências Biológicas Universidade Federal de Minas Gerais—UFMG Belo Horizonte Brazil

**Keywords:** dogs, efficacy, leishmaniasis, protection, zoonosis

## Abstract

Canine visceral leishmaniasis (CVL) is a zoonotic disease of great worldwide importance and can be prevented by vaccinating seronegative dogs. The objective of the present systematic review and meta‐analysis is to verify the efficacy rate of vaccines tested in dogs against CVL or *L. infantum* infection. We used PRISMA guidelines for this review and Pubmed, Web of Science, Scopus, Cochrane, Scielo and CABI to find studies about vaccines against CVL in dogs. Articles were analysed and grouped according to the antigens used. The risk of bias analysis was performed using SYRCLE's RoB tool and meta‐analysis using R Statistical language. The final analysis was conducted using 22 studies that assessed DNA, excreted/secreted proteins and subunit vaccines, involving a total of 92 animals, 96 animals and 78 animals, respectively. Regarding DNA vaccines, the analyses revealed non‐significant results in terms of preventing parasite presence in the organs or the onset of clinical signs. However, subunit vaccines demonstrated statistically significant results concerning parasite presence in the organs, but not when it comes to clinical signs. Additionally, there was no statistically significant difference observed in parasite burden in the organs or clinical signs for the excreted/secreted vaccines. The meta‐analysis indicated that subunit and excreted/secreted protein vaccines were significantly more effective in preventing parasites in vaccinated animals compared to both DNA‐based vaccines and control groups. Heterogeneity among studies is a limitation, emphasising the need for standardised protocols for reliable comparisons.

## 1 Introduction

Leishmaniasis is a complex of neglected diseases present in 98 countries in Europe, Africa, Asia and America, caused by obligate intracellular protozoa of the genus *Leishmania*, which infect an average of 0.9–1.7 million people every year [1]. In Central and South America, the most common species, called *L. infantum*, is commonly transmitted from animals to humans by the bite of the female sandfly *Lutzomyia longipalpis* [2]. It is considered a public health problem since the number of cases of the disease in dogs is directly related to the number of human cases of leishmaniasis because the parasite, residing in the skin of animals, is also transmitted vectorially to people [3].

Dogs are considered the main reservoirs of the disease in an urban environment and may show clinical signs such as weight loss, lymphadenopathy, skin lesions, onychogryphosis, muscle atrophy and ocular signs [4]. It is also common for there to be the existence of resistant dogs, which do not show clinical signs, called asymptomatic, however, even so, they are a source of infection for the sandfly [5]. The resistance of these dogs can be attributed to the actions of the immune system against the parasite. The Th1 immune response profile, in which there is stimulation of a response mediated by TCD4+Th1 and TCD8+ lymphocytes, is associated with lower development of clinical signs in animals, with the participation of natural killer cells, with high production of IL‐12, IL‐2, IFN‐γ and TNF‐α and lower production of IL‐10 [6]. This environment is conducive to the classical activation of macrophages, which will produce oxygen and nitric oxide metabolites, responsible for destroying the protozoan inside the cell [7]. Contrary to what occurs in animals whose predominant profile is Th2, characterised by higher amounts of IL‐4, IL‐5, IL‐10 and TGF‐β and cells with a lower capacity to destroy the protozoan and, therefore, responsible for a greater susceptibility to infection and the appearance of clinical signs [8].

CVL can be prevented by controlling the vector population, individual protection measures like 4% deltamethrin repellent collars, preventing the movement of animals in environments where sandflies can inhabit, especially at twilight and vaccination [8, 9]. The vaccination is considered an individual protection measure because the vaccines available on the market protect against the disease but not against infection in animals [10]. For this reason, it is necessary to develop a new vaccine antigen that protects against disease but also infection, since decreasing the number of infected dogs, the probability of transmission of the parasite to the sandfly is also reduced and consequently the number of human cases of leishmaniasis. Therefore, it is necessary to have a vaccine that stimulates immune mechanisms linked to animal resistance, in addition to meeting ideal characteristics such as a smaller number of applications and a lower cost.

However, due to the complexity of the protozoan, this objective is still challenging, with only two vaccines available commercially in the world, which present partial protection against CVL and do not represent a significant impact on the reduction of human cases of Leishmaniasis, since they do not prevent infection in dogs [10]. Therefore, there are several studies aimed at the development of effective immunogens against CVL and the infection of animals by *L. infantum*, but many of them have not reached satisfactory levels of protection or need more adequate protocols to achieve these purposes. The objective of this systematic review and meta‐analysis is to verify the efficacy of vaccines against CVL or the parasite *L. infantum*, tested in dogs, to demonstrate which of them achieve the desired protection and which are the best protocols to be used.

## 2 Methods

This study was registered in the Prospective Register of Systematic Reviews (PROSPERO, ID: CRD42022322176). The guidelines of the PRISMA statement (Preferred Reported Items for Systematic Reviews and Meta‐Analysis) were adopted in this review (Table S1).

### 2.1 Strategy of Search and Selection of the Studies

The review started with searches for studies in Pubmed, Web of Science, Scopus, Cochrane, Scielo and CABI databases on 9 September 2020, and the terms were searched by a reviewer (J.A.M.P.) in the title, abstract and text sections. The PICOT (population, intervention, comparison, outcome and time) used for the searches, which involved the canine population, the different types of vaccines against CVL used for prophylaxis, their efficacy and protection, is described in the Table S2.

Initially, the studies found in the databases were added to a reference management software, where they were selected by title by two reviewers (J.A.M.P. and T.F.M.), who also independently selected the studies by abstract. Titles that contained information about *Leishmania* species other than *L. infantum* or other host species other than the canine were excluded, as it was the case in the selection of studies by abstract. In a subsequent step, the full texts were analysed by two reviewers (J.A.M.P. and T.F.M.) and included or excluded based on predetermined criteria. Disagreements between the two reviewers were resolved by a third reviewer (E.M.S.D.).

On 9 April 2024, a search update was conducted on the databases using the same terms, with a filter applied to restrict results between the years 2020 and 2024. Two reviewers (J.A.M.P. and A.L.G.O.) independently selected articles based on pre‐established criteria, as done previously.

### 2.2 Inclusion and Exclusion Criteria

The papers included in the review were those that fit the following criteria: (i) published in all countries, (ii) published in all years, (iii) that talked about vaccines used for prophylaxis, (iv) against canine visceral leishmaniasis (CVL), (v) in dogs, (vi) *L. infantum* species. Studies in languages other than English, Spanish or Portuguese and that fitted the exclusion criteria detailed in Table S3 were excluded. This step was performed by two reviewers (J.A.M.P. and T.F.M.).

### 2.3 Risk of Bias Analysis Using SYRCLE's RoB Tool

After the inclusion of the papers by pre‐established criteria, an internal validation was carried out by a specific protocol, created to analyse the risks of bias in studies with animals, called SYRCLE's RoB tool [11]. This protocol consists of evaluating five different types of bias: (i) selection bias, (ii) performance bias, (iii) detection bias, (iv) attrition bias, (v) reporting bias and (vi) other biases. These five types of bias are divided into 10 questions or domains that must be answered with ‘Yes’, ‘Unclear’ or ‘No’ which respectively mean low, uncertain or high risks of bias.

### 2.4 Type of Studies

Only original studies were included. Trials such as cohort, case–control, cross‐sectional, case series, case reports and reviews were excluded.

### 2.5 Data Extraction

Data were extracted by one of the reviewers (J.A.M.P.) and verified by two reviewers (M.M.O. and E.M.S.D.). The author, the year of publication, the country where the study was carried out and the type of vaccine antigen tested were first extracted. Subsequently, the characteristics of the evaluated groups were extracted, such as age, sex, the total number of animals per group, breed, dose, number of vaccinations, time between doses, type of adjuvant and route of administration. As for the animals in the control group, the number of animals and what was applied to them, the period, dose, and route of the experimental challenge and the period of exposure to the parasite were extracted. For studies that performed natural challenges, the diagnostic methods used to confirm infection as well as tests performed to verify the immunogenicity of the antigen, were the data extracted from each study for further analysis. The same dataset of articles published between 2020 and 2024 was extracted in the 2024 update by two reviewers (J.A.M.P. and A.L.G.O.).

### 2.6 Data Synthesis and Analysis

For the meta‐analysis, the trials were initially categorised based on the method of infection in animals, either natural or experimental. They were then further divided into groups based on the types of vaccines tested, which included DNA vaccines, subunit vaccines and vaccines composed of secreted/excreted proteins. Despite being produced differently, subunit vaccines and vaccines composed of secreted/excreted proteins are both made up of proteins and therefore form a fourth group. The raw data extracted from the trials that made up each group, for the meta‐analysis, were related to the number of animals in the vaccinated (those who received one or more doses of the vaccine) and non‐vaccinated groups (those who received a placebo), who exhibited clinical signs (Outcome 1) and parasite load in any analysed organ (Outcome 2).

The values of overall effect (*Z*) were used to calculate vaccine efficacy. For there to be a statistically significant difference between the vaccinated and non‐vaccinated groups, indicating that the vaccines in the group were effective, a *p* ≤ 0.05 was considered. To assess the heterogeneity of the trials, the *I* ^2^ was calculated, with low heterogeneity defined as *I* ^2^ ≤ 50%, moderate heterogeneity as *I* ^2^ between 50% and 70%, and high heterogeneity as *I* ^2^ > 70%. Also, the relative risk (RR) and the confidence interval (CI) were calculated [12].

The analysis was done using the R Statistical language (v4.3.1) on Windows 11 × 64, using the packages netmeta (v2.9.0), meta (v7.0.0), Matrix (v1.6.1), data.table (v1.14.8), numDeriv (v2016.8.1.1), MAd (v0.8.3), report (v0.5.8.5), dlookr (v0.6.3), metaforest (v0.1.4), metafor (v4.6.0), metadat (v1.2.0), ggplot2 (v3.5.0), dplyr (v1.1.2), ranger (v0.16.0) and markdown (v1.12) [13].

## 3 Results

### 3.1 Selected Studies

**FIGURE 1:**
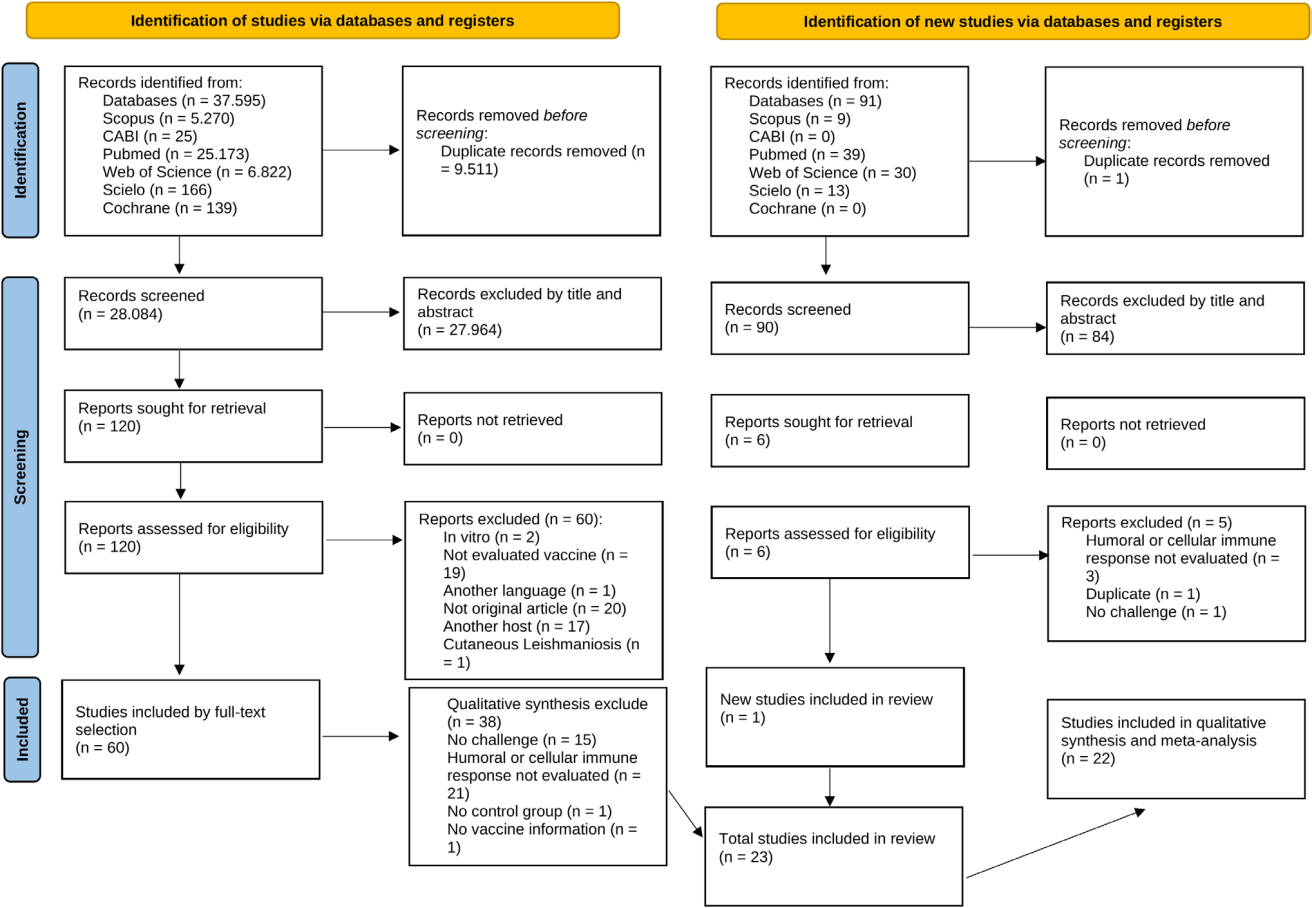
Flow chart of the search and selection of studies for the systematic review of the efficacy of vaccines against CVL.

In the initial search, 37,595 studies were found and, among them, 9,507 duplicates were detected by the reference management software, totaling 28,088 articles that were included in the initial selection by titles (Figure 1). The studies that contained the words ‘Canine’, ‘*Leishmania*’ or ‘Vaccine’ were kept for the selection of abstracts, also carried out using the same criteria. In the end, 76 papers were selected to be analysed by quality criteria, and 22 were eligible for the final analyses and risk of bias analysis by SYRCLE's RoB Tool (Table 1). All these studies were carried out between 2003 and 2020. Yet, an update of the review was conducted through a new search in the databases using the same terms, limiting the publication time of studies between 2020 and 2024. This search resulted in 91 articles. Of these, 90 were excluded after abstract review. Only one article was included in the review and submitted to the risk of bias analysis by SYRCLE's RoB Tool (Figures S1 and S2). However, this article presented its data summarised in graphs and therefore was not included in the meta‐analysis. The 77 articles excluded after the selection stage by eligibility criteria, as well as the reasons why they were not included in this review, are shown in Table S4. Therefore, the initial 22 articles that were included were retained for the meta‐analysis. Studies that performed more than one experiment, analysed different antigens, or the same antigen at different times, compared to more than one control group, or with different challenge characteristics, such as time or dose, were defined as trials. In the end, 45 trials were included in this meta‐analysis.

**TABLE 1:** General characteristics of studies of vaccines against CVL included in this systematic review.

First author, year	Country	Type of study	Total number of animals	Dog breeds	Sex	Age
Abbehusen, 2018^a^ [14]	Brazil	Intervention^d^	30	Beagle	Both^e^	2–3 m^**f**^
Aguiar‐Soares, 2014 [15]	Brazil	Intervention^d^	20	UB	Both^e^	7–8 m^**f**^
Alcolea, 2019 [16]	Spain	Intervention^d^	10	Beagle	Both^e^	12–18 m^**f**^
Alonso, 2023 [17]	Spain	Intervention^d^	30	Beagle	Both^e^	12–18 m^**f**^
Borja‐Cabrera, 2009^b^ [18]	Brazil	Intervention^d^	19	UB	Both^e^	4 m^**f**^
Bourdoiseau, 2009^b^ [19]	France	Intervention^d^	12	UN	Both^e^	UN
Carcelen, 2009 [20]	Spain	Intervention^d^	21	Beagle	Both^e^	12–24 m^**f**^
De Lima, 2010 [21]	Iran	NC	40	UN	Both^e^	UN
Fernandes, 2008 [22]	Brazil	Intervention^d^	21	Beagle	Both^e^	3–9 m^**f**^
Fiuza, 2015 [23]	Brazil	Intervention^d^	18	Beagle	Both^e^	8 m^**f**^
Gradoni, 2005 [24]	Brazil	NC	45	Beagle	Both^e^	6 m^**f**^
Lemesre, 2005 [25]	France	Intervention^d^	18	Beagle	Both^e^	12–72 m^**f**^
Lemesre, 2007 [26]	France	NC	414	UN	Both^e^	UN
Martin, 2014 [27]	France	Intervention^d^	20	Beagle	Both^e^	6 m^**f**^
Petitdidier, 2016 [28]	France	Intervention^d^	19	Beagle	Both^e^	24–48 m^**f**^
Petitdidier, 2019 [29]	France	Intervention^d^	15	Beagle	Both^e^	24–48 m^**f**^
Poot, 2009 [30]	Germany	Intervention^d^	44	Beagle	Both^e^	6 m^**f**^
Poot, 2006 [31]	France	Intervention^d^	15	Beagle	Males	6 m^**f**^
Ramiro, 2003 [32]	Spain	Intervention^d^	20	Beagle	Both^e^	18–54 m^**f**^
Roatt, 2012 [33]	Brazil	Intervention^d^	20	UB	Both^e^	UN
Rodriguez‐Cortés, 2007 [34]	Spain	Intervention^d^	12	Beagle	Females	9 m^**f**^
Shahbazi, 2015 [35]	Iran	Intervention^d^	30	UB	Both^e^	6–48 m^f^
Velez, 2020^c^ [36]	Spain	NC	168	UN	Both^e^	> 6^g^ m^f^

Abbreviations: NC, Natural challenge; UB, Undefined breed; UN, Uninformed.

^a^ This study has been corrected and its errata were also considered in this review.

^b^ Only some data were taken from this study, which fit the quality criteria.

^c^ In this article, the number of animals considered were the ones that ended up as vaccinations and did all the tests.

^d^ Studies that performed experimental infection in dogs.

^e^ Both sexes.

^f^ m: age defined in months.

^g^ > 6: more than 6 months.

### 3.2 Vaccine, Dose and Route

Of the selected studies, 82.6% (19/23) underwent experimental infection and only 17.4% (4/23) performed a natural challenge, in which the evaluated animals were exposed to the parasite in the environment in which they lived. Individuals of both sexes were used in 91.3% (21/23) of the studies, and only one article [34] used only females in their groups, with the number of dogs per group varying between three and 85 and ages ranging from 2 to 54 months. The vaccinated and control groups were predominantly composed of beagle animals, used in 65.2% (15/23) of the studies.

In most studies, 65.2% (15/23) tested vaccines composed of proteins or peptides (second‐generation vaccines) and 34.8% (8/23) of tested vaccines composed of genetic material (third‐generation vaccines). However, among those selected in this review, there were no articles that tested first‐generation vaccines, consisting of the inactivated or attenuated protozoan.

The studies were divided into trials for meta‐analysis and used different antigens. The most tested antigens among the selected trials were the so‐called *L. infantum* secreted/excreted antigens (LiESAp), together with MDP adjuvants [19, 25], QA‐21 [27] or saponin [36]. The efficacy of Q protein [20], recombinant A2 antigen [22] and FML [21, 23] was also tested. *Leishmania* antigen‐activated C‐kinase antigen (LACK) has been tested in different ways by different studies [16, 17, 32, 34], as well as many others presented in Table 2. Only 4.44% (2/45) trials applied only one dose of its immunogen [20, 23] and only 2.22% (1/45) [34] performed four applications. Most trials performed two or three applications, with intervals varying between 15 and 28 days (Table S6) and the most common route of administration was subcutaneous, used in 88.9% (40/45) of the time (Table S5).

**TABLE 2:** Vaccination and challenge data from trials selected for systematic review and meta‐analysis^a^.

First author, year	Vaccine's generation	N Vac	N C	Control group	Vaccination			Challenge				Time (days)
					Antigen/adjuvant/antigen dose	Age (months)	Route	Type of challenge	Strain	Dose	Route	
Abbehusen, 2018	3a	10	10	P	LJM17 (250 μg/10^8^)	2–3	IM	E	LIP	10^7^	ID	30
Abbehusen, 2018	3a	10	10	P	LJL143 (250 μg/10^8^)	2–3	IM	E	LIP	10^7^	ID	30
Aguiar‐Soares, 2014	2a	5	5	P	LB + SGE/Saponin (5 acini)	7–8	SC	E	LIP	10^7^	ID	105
Aguiar‐Soares, 2014	2a	5	5	P	LB + SGE (5 acini + 600 μg)	7–8	SC	E	LIP	10^7^	ID	105
Aguiar‐Soares, 2014	2a	5	5	P	LB + SGE/Saponin (5 acini + 600 μg)	7–8	SC	E	LIP	10^7^	ID	105
Alcolea, 2019	3a	5	5	P	pPAL‐LACK (200 μg)	12–18	IN	E	LIP	10^8^	UN	60
Borja‐Cabrera, 2009	3a	6	13	P	VR1012‐NH36 (750 μg)	4	IM	E	LIA	7 × 10^8^	UN	25
Bourdoiseau, 2009	2a	3	3	A	LiESAp/MDP (100 μg)	Not informed	SC	E	LIP	10^8^	IV	63
Bourdoiseau, 2009	2a	3	3	A	LiESAp/MDP (100 μg)	Not informed	SC	E	LIP	10^8^	IV	252
Carcelen, 2009	2a	7	7	P	Q Protein (100 μg)	12–24	SC	E	LIP	10^5^	IV	60
Carcelen, 2009	2a	7	7	P	Q Protein (100 μg)	12–24	SC	E	LIP	10^5^	IV	39
Lemesre, 2005	2a	3	3	A	LiESAp/MDP (100 μg)	12–72	SC	E	LIP	10^8^	IV	84
Lemesre, 2005	2a	3	3	A	LiESAp/MDP (100 μg)	12–72	SC	E	LIP	10^8^	IV	84
Lemesre, 2005	2a	3	3	A	LiESAp/MDP (200 μg)	12–72	SC	E	LIP	10^8^	IV	84
Lemesre, 2005	2a	3	3	A	LiESAp/MDP (50 μg)	12–72	SC	E	LIP	10^8^	IV	84
Martin, 2014	2a	10	10	P	LiESP/QA‐21 (100 μg)	6	SC	E	LIP	10^8^	IV	365
Fernandes, 2008	2a	7	7	P	rA2/Saponin (100 μg)	3–9	SC	E	LIP	5 × 10^7^	IV	28
Fiuza, 2015	2a	6	6	P	LdCen (1 × 10^7^)	8	SC	E	LIP	10^7^	IV	
Fiuza, 2015	2a	6	6	P	FML (1.5 mg)	8	SC	E	LIP	10^7^	IV	
Petitdidier, 2016	2a	9	5	P	LaPSA‐38S/QA‐21 (25 μg)	24–48	SC	E	LIP	10^8^	IV	60
Petitdidier, 2016	2a	5	5	P	LaPSA‐12S/QA‐21 (25 μg)	24–48	SC	E	LIP	10^8^	IV	60
Petitdidier, 2019	2a	10	5	P	A17G + A17E + E34PC/QA‐21 (25/25/10 μg)	24–48	SC	E	LIP	10^8^	IV	120
Poot, 2006	2a	5	5	P	rCPA + rCPB/rIL‐12 (50/50/10 μg)	6	SC	E	LIP	5 × 10^7^	IV	
Poot, 2006	2a	5	5	P	rCPA + rCPB/rIL‐12 + QuilA (50/50/1 μg)	6	SC	E	LIP	5 × 10^7^	IV	
Poot, 2009	2a	7	7	P	rJPCM5_Q/MDP (70 μg)	6	SC	E	LIP	5 × 10^7^	IV	28
Poot, 2009	2a	7	7	P	rJPCM5_Q/aluminium hydroxide (70 μg)	6	SC	E	LIP	5 × 10^7^	IV	28
Poot, 2009	2a	7	7	P	rJPCM5_Q/ISCOMatrix C (70 μg)	6	SC	E	LIP	5 × 10^7^	IV	28
Poot, 2009	2a	5	1	P	rJPCM5_Q/MDP (70 μg)	6	SC	E	LIP	5 × 10^7^	IV	21
Poot, 2009	2a	5	1	P	rJPCM5_Q/aluminium hydroxide (70 μg)	6	SC	E	LIP	5 × 10^7^	IV	21
Poot, 2009	2a	5	1	P	rJPCM5_Q/ISCOMatrix C (70 μg)	6	SC	E	LIP	5 × 10^7^	IV	21
Ramiro, 2003	3a	5	5	P	DNA‐LACK (100 μg)	18–54	SC	E	LIP	10^8^	IV	0
Ramiro, 2003	3a	5	5	P	DNA‐LACK + rVV‐LACK (10^8^ pfu)	18–54	SC	E	LIP	10^8^	IV	0
Roatt, 2012	2a	5	5	P	LBf/Saponin (600 μg)	UN	SC	E	LIP	10^7^	IV	105
Rodriguez‐Cortés, 2007	3a	6	6	P	pMOK‐Kmp11/‐TRYP/‐LACK/‐GP63 (200 μg)	9	ID	E	LIP	5 × 10^7^	IV	30
Shahbazi, 2015	3a	10	10	P	pcDNA‐A2‐CPACPB−CTEGF P (cSLN) (200 μg)	6–48	SC	E	LIP	4 × 10^7^	IV	42
Shahbazi, 2015	3a	10	10	P	pcDNA‐A2‐CPACPB−CTEGFP (electroporation) (200 μg)	6–48	SC	E	LIP	4 × 10^7^	IV	42

Abbreviations: 2a, second generation; 3a, third generation; A, adjuvant; Dose, unity = parasites; E, experimental challenge; ID, intradermal; IM, intramuscular; IN, intranasal; IV, intravenous; LIA, *L. infantum* amastigotes; LIP, *L. infantum* promastigotes; N C, number of control animals; N Vac, number of vaccinated animals; P, placebo; rJPCM5_Q, antigen produced by Baculovirus; rJPCM5_Q, antigen produced by *E. coli* ; SC, subcutaneous; UN, uninformed; V, empty vector.

^a^ Only studies with comprehensive vaccination and challenge data were included in this table. Trials with missing or incomplete information were excluded from this summary.

### 3.3 Methods to Confirm Immunogenicity

To confirm the immunogenicity of the antigens, the studies carried out several tests. To assess cellular immune response, 10.5% (2/19) of the studies that performed experimental infection used the Enzyme‐linked immunosorbent assay (ELISA) and 57.9% (11/19) used RT‐qPCR to evaluate cytokines such as IL‐10, IFN‐γ and IL‐4 in materials such as animal serum and cell culture supernatant. For the evaluation of cell types present in culture, flow cytometry was used in 36.84% (7/19) of the studies. 26.3% (5/19) studies also evaluated the production of nitric oxide (NO) and the canine macrophage leishmanicidal assay (CMLA). For analysis of humoral immune response, antibody titers in animal sera were evaluated through ELISA tests, used in 100% of experimental infection trials (Table S7). All studies of natural infection (Table S8) used ELISA for evaluating antibody titers and 50% (2/4) performed IFAT [20, 27].

### 3.4 Challenge Strains, Dose and Route of Exposure

To calculate the efficacy rates of the tested vaccines, the studies conducted experimental or natural challenges on their vaccinated and control groups. All experimental infection studies used promastigotes of *L. infantum* in the challenge, in more common doses of 5 × 10^7^ parasites, in 28.9% (13/45) of the trials, 10^8^ parasites, in 26.7% (12/45) of the trials, and 10^7^ parasites, in 17.8% (8/45) of the trials. The most used route to perform the challenge was intravenous, and the periods ranged from 0 to 240 days after the last vaccination. Among those studies that performed natural infection, the exposure periods varied between 30 and 720 days after the last dose of immunogen was administered to the animals.

### 3.5 Diagnostic Methods to Confirm Infection and Evaluation of Clinical Signs

To confirm the presence or absence of infection, the following tests were performed in the studies: qPCR testing in 78.26% (18/23), ELISA in 21.74% (5/23), direct tissue visualisation testing in 17.39% (4/23), parasite culture in 47.82% (11/23), IFAT in 8.69% (2/23) and DAT in 4.34% (1/23). Clinical evaluation was conducted to detect infected, symptomatic or asymptomatic animals; this evaluation was performed in 65.21% (15/23) of the studies. The main signs observed were skin and adnexal lesions (alopecia, ulcers, exfoliative dermatitis, onychogryphosis), nutritional status, eye lesions (uveitis, conjunctivitis, keratoconjunctivitis) and lymphadenopathy. These changes are commonly found in dogs with CVL.

### 3.6 Statistical Difference Between Vaccinated and Non‐Vaccinated Animals in Meta‐Analysis

**FIGURE 2:**
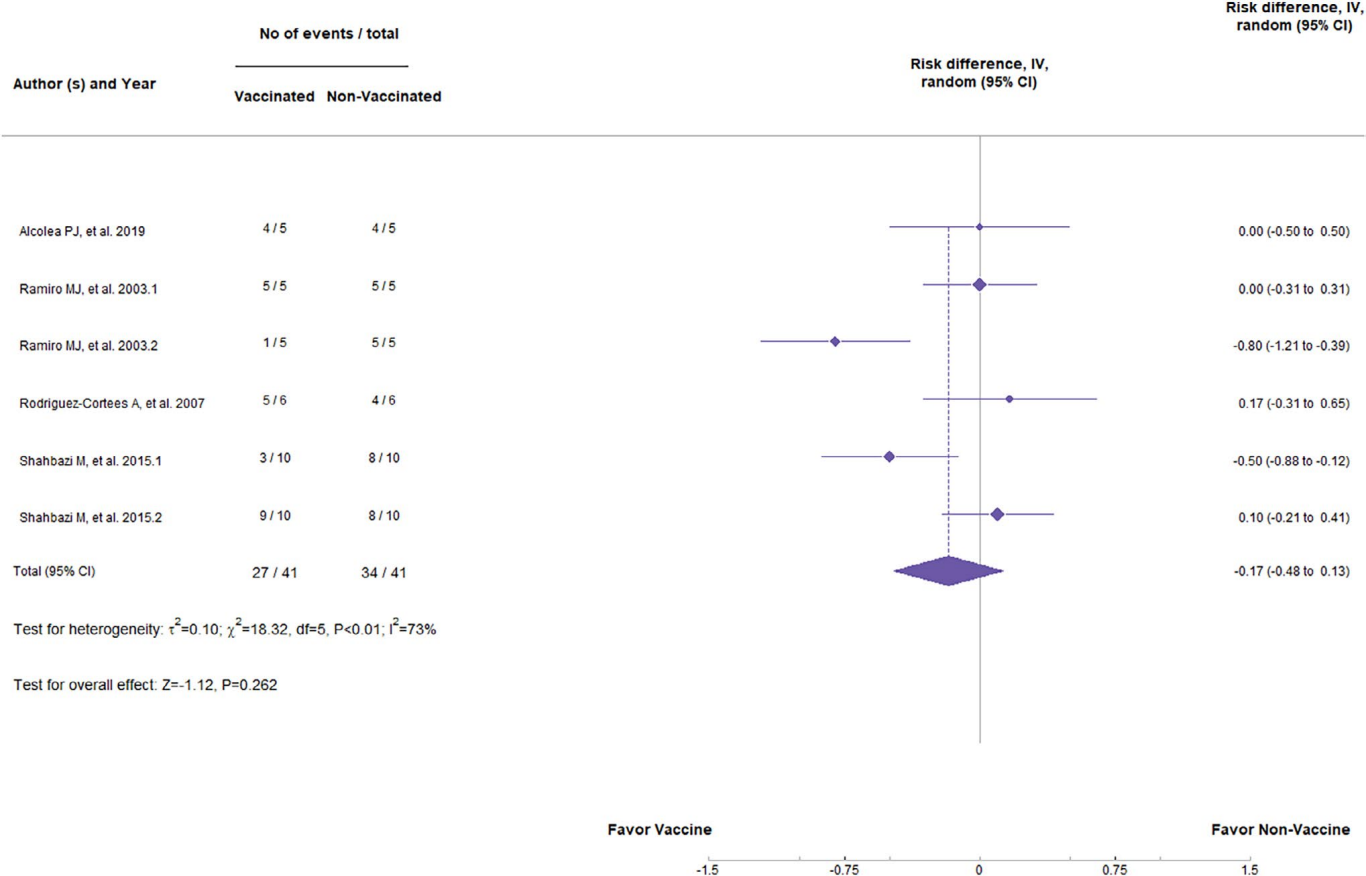
Forest plot showing the statistical difference of DNA vaccines in preventing clinical manifestations of canine visceral leishmaniasis (CVL).

**FIGURE 3:**
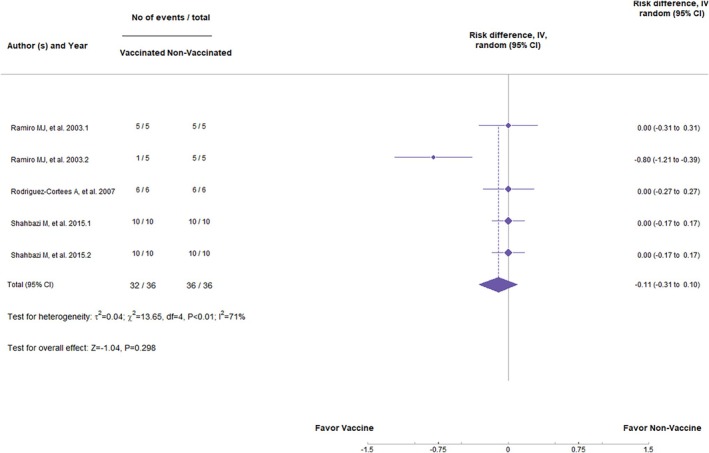
Forest plot showing the statistical difference of DNA vaccines in preventing parasite load.

**FIGURE 4:**
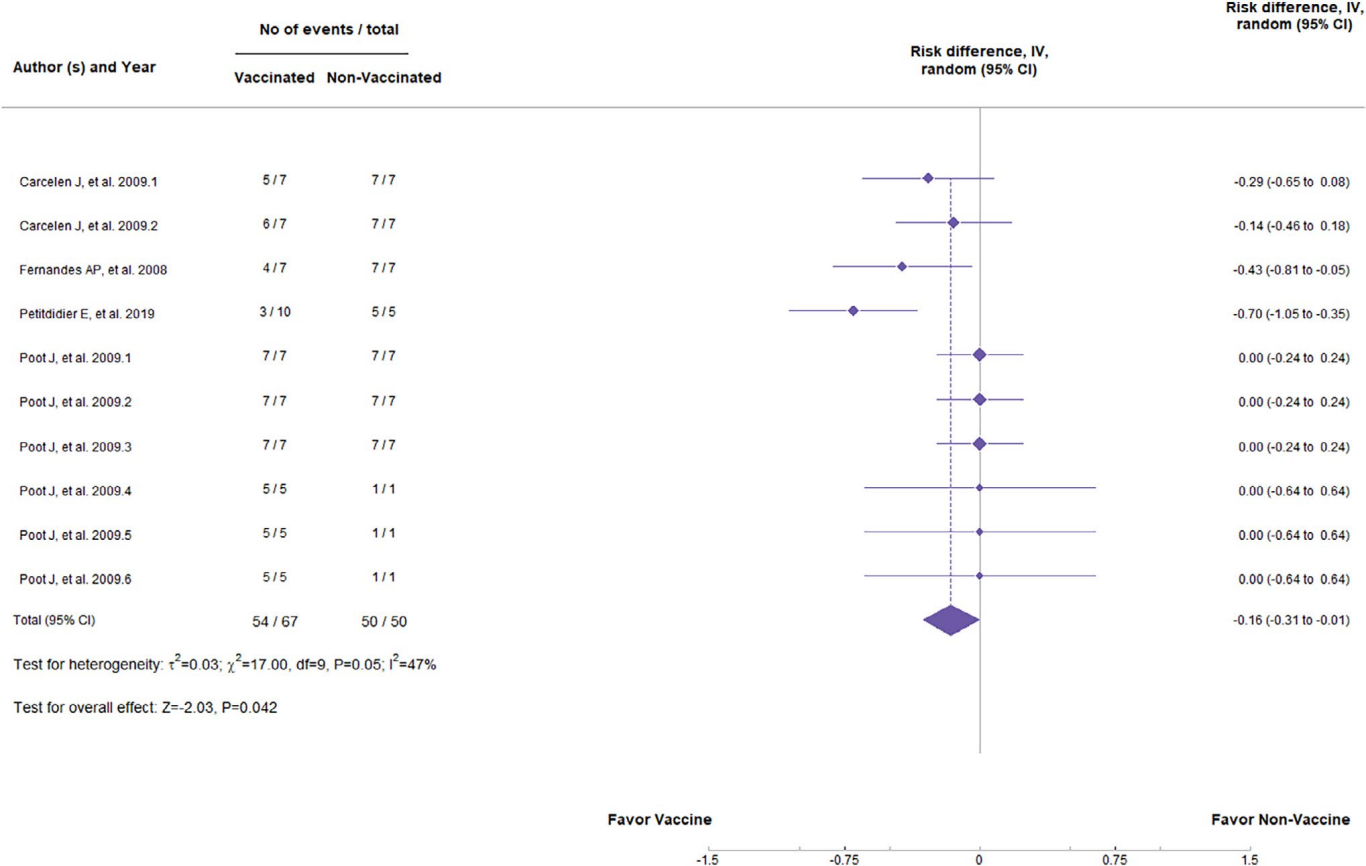
Forest plot showing the statistical differences of subunit vaccines in preventing parasite load.

**FIGURE 5:**
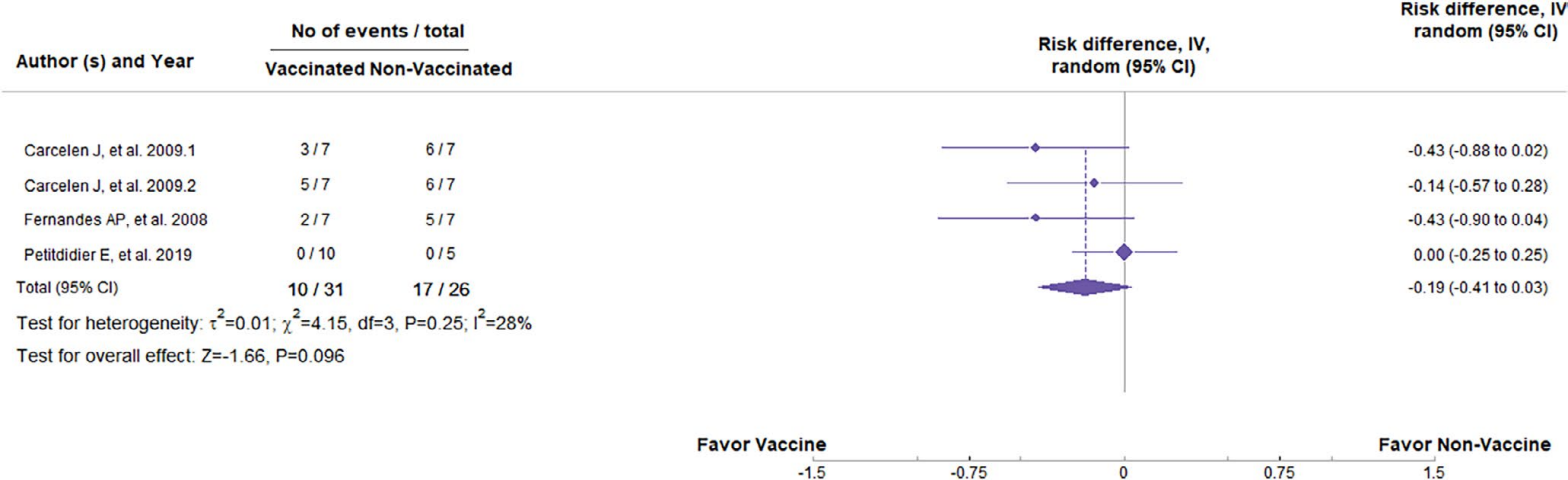
Forest plot showing the statistical difference of subunit vaccines in preventing clinical manifestations of canine visceral leishmaniasis (CVL).

**FIGURE 6:**
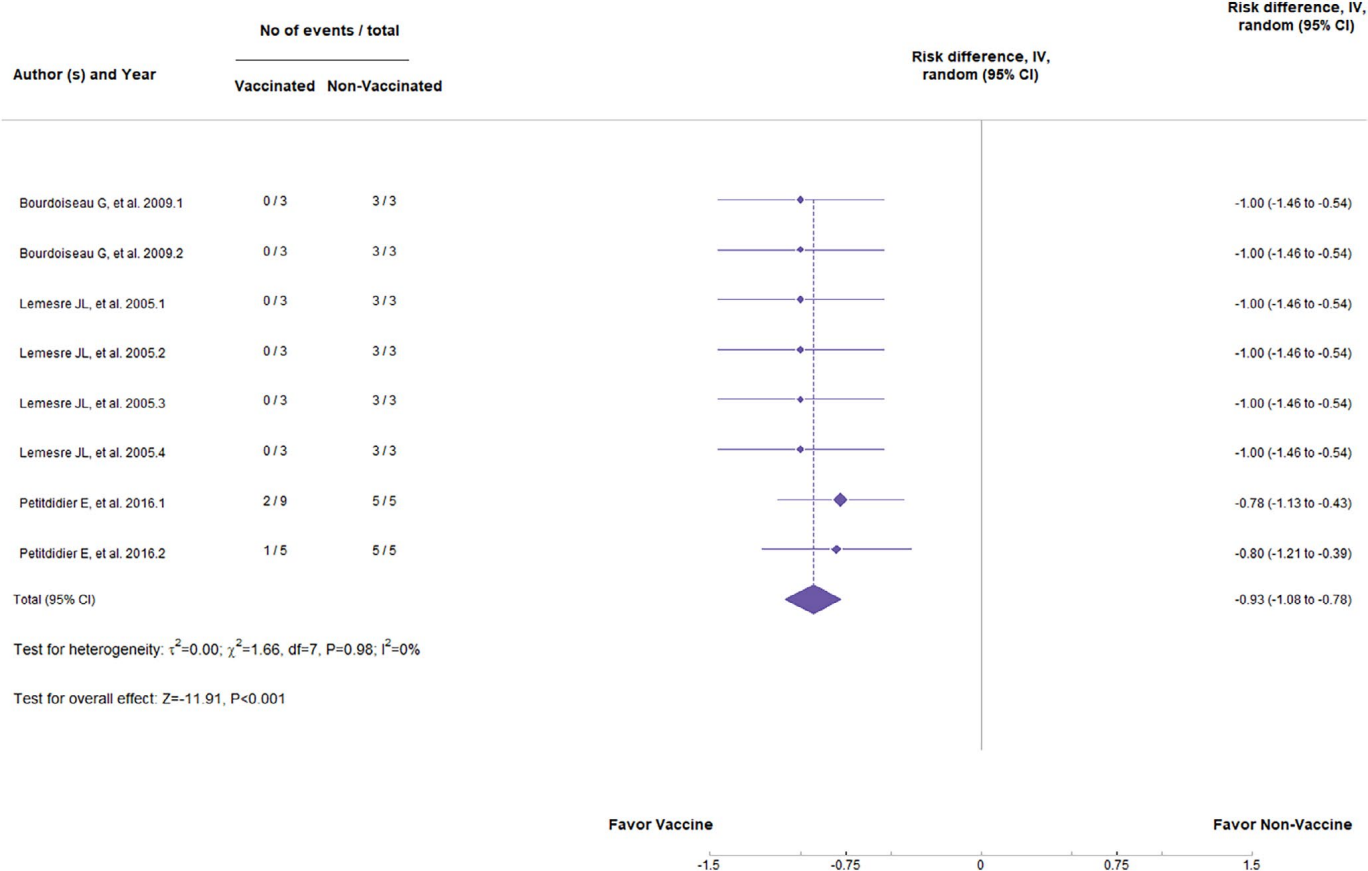
Forest plot showing the statistical difference of secreted/excreted proteins vaccines in preventing parasite load.

**FIGURE 7:**
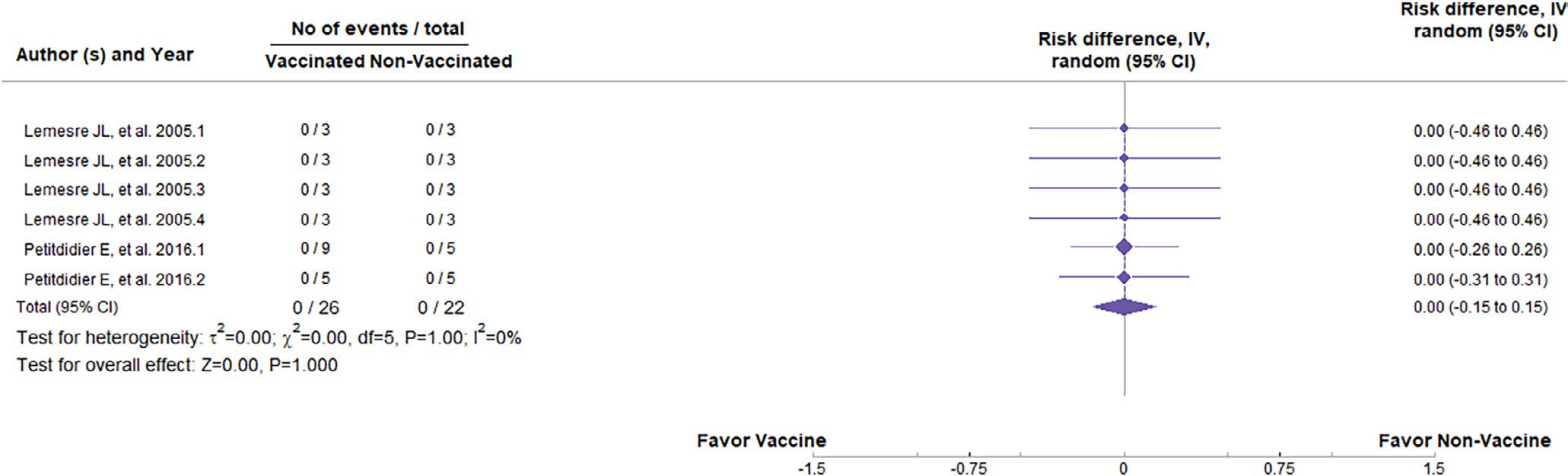
Forest plot showing the statistical difference of secreted/excreted proteins vaccines in preventing clinical manifestations of canine visceral leishmaniasis (CVL).

DNA vaccines, in general, have had no significant statistical difference in preventing clinical signs (*Z* = −1.12, *p* = 0.262; RR = −0.17, 95%, *p* < 0.01, CI: −0.48 to 0.03) (Figure 2) or the appearance of parasites in the organs (*Z* = −1.04, *p* = 0.298; RR = −0.11, 95%, *p* < 0.01, CI: −0.31 to 0.10) (Figure 3). Values of *I* ^2^ = 73% and 71%, respectively, indicate high heterogeneity among the studies, suggesting considerable variation between the trials. On the other hand, the subunit vaccines showed a statistically significant difference in preventing parasite load in the organs (*Z* = −2.03, *p* = 0.042; RR = −0.16, 95%, *p* = 0.05; CI: −0.31 to −0.01) (Figure 4). Besides that, they did not demonstrate significant statistical differences in clinical signs (*Z* = −1.66, *p* = 0.096; RR = −0.19, 95%, *p* = 0.25, CI: −0.41 to 0.03) (Figure 5). However, *I* ^2^ = 28% and *I* ^2^ = 47% indicate low heterogeneity among the studies. The same occurred with excreted/secreted proteins (ESPs). The analysis showed a *Z* = −11.91 (*p* < 0.001; RR = −0.93, 95%, *p* = 0.98, CI: −1.81 to −0.78) for the parasite load (Figure 6) and *Z* = −0.00 (*p* = 1.00; RR = 0.00, 95%, *p* = 1.00, CI: −0.15 to 0.15) for the clinical signs (Figure 7). We can conclude that vaccines were effective in preventing the appearance of parasites in the organs but not in clinical signs. The value of *I* ^2^ = 0% in each analysis indicates high homogeneity between trials.

## 4 Discussion

This systematic review and meta‐analysis aimed to determine whether there are statistically significant differences in the occurrence of clinical signs of CVL and parasite burden in organs between vaccinated and non‐vaccinated animals with different antigenic groups tested as vaccines. Our results suggest a statistically significant trend that animals vaccinated with subunit vaccines are less susceptible to the presence of parasites in organs compared to non‐vaccinated animals (*Z* = −2.03, *p* = 0.042), thus having a lower risk of developing *Leishmania* infection (RR = −0.16, 95% CI: −0.31 to −0.01, *p* = 0.05). This suggests that subunit vaccines may be more effective in inducing a lasting immune response and controlling parasite burden.

Regarding the development of clinical signs, there was no significant difference between vaccinated and non‐vaccinated animals (*Z* = −1.66, p = 0.096; RR = −0.19, 95% CI: −0.41 to 0.03, *p* = 0.25); however, the *Z* and RR values were favourable to vaccine administration, as shown in Figure 5. For vaccines composed of ESPs, the results were promising. Statistically, vaccinated animals tended to be less susceptible to parasite burden (*Z* = −11.91, *p* < 0.001; RR = −0.93, 95% CI: −1.81 to −0.78, *p* = 0.98). However, there was no statistical difference in terms of clinical signs (*Z* = −0.00, *p* = 1.00; RR = 0.00, 95% CI: −0.15 to 0.15, *p* = 1.00). Nevertheless, the analysis of DNA vaccines did not show significant differences in any of the outcomes, despite the favourable values towards vaccine administration, indicating that they may not be immunogenic enough to induce a protective immune response.

It is important to highlight that ESP vaccines demonstrated greater protective capacity compared to other types of vaccines. These proteins are components released by the parasite into the extracellular environment and play a role in the host–parasite interactions.

The superior efficacy of ESPs‐based vaccines may be attributed to their high immunogenicity, which is rapidly recognised by the host immune system, triggering a predominantly Th1 response [37]. In dogs, a protective immune response is characterised by the activation of CD4^+^ Th1 cells, which secrete pro‐inflammatory cytokines such as IFN‐γ, TNF‐α and IL‐12, promoting macrophage activation and nitric oxide (NO) production [37]. Markikou‐Ouni, Drini [38] showed that ESPs immunisation increases dendritic cell maturation and antigen presentation, leading to robust IFN‐γ production and reduced parasite loads in target organs such as the spleen, liver and bone marrow. These parasite‐clearing mechanisms result in improved infection control. Another important aspect is that ESPs‐derived antigens interact with toll‐like receptors (TLRs) and with pathogen‐associated molecular pattern (PAMP) receptors, stimulating innate immune activation [38]. This process stimulates the production of IL‐12 and IFN‐γ, amplifying the protective Th1‐type response [38].

It is important to note that ESPs can act as more efficient immunogens than conventional parasite antigens. However, the selection of ESPs for vaccine formulation should consider not only their immunogenic potential, but also their effects on modulating the immune response. Some ESPs, such as GP63, can interact with antigen‐presenting cells (APCs), but due to their ability to downregulate MHC‐II expression, they can impair antigen presentation [38]. This process can decrease the activation of CD4^+^ Th1 T cells, allowing the parasite to persist despite partial immune activation. These proteins can also stimulate regulatory mechanisms, since the production of IL‐10 and TGF‐β varies depending on their protein composition, influencing the immune response in different ways [38]. For this reason, it is necessary to better characterise ESPs to identify the fractions that contribute to the polarisation of Th1‐type responses in dogs [39]. Combining these antigens with adjuvants that enhance the protective response, such as monophosphoryl lipid A (MPL‐A) and CpG, can significantly improve vaccine performance. These adjuvants stimulate the TLR4 and TLR9 pathways, respectively, leading to increased IFN‐γ production and a more effective Th1 response [40]. Furthermore, ESPs can contribute to the development of long‐lasting adaptive immunity by activating CD4^+^ and CD8^+^ T lymphocytes. Increased IFN‐γ and IL‐12 production helps establish the Th1 response profile, leading to efficient parasite control and improved disease control in endemic areas [37]. Despite the strong immunogenicity of ESPs in dogs, their introduction as a preventive measure still faces significant limitations. One of the main concerns is the difficulty in differentiating infections from vaccinated animals in endemic regions such as Brazil. Since these proteins are naturally present during infection, widely used serological tests can produce false‐positive results in vaccinated animals, leading to diagnostic errors that can complicate disease control and treatment decisions. Furthermore, the variability in the composition of ESPs, depending on the stage of the parasite and how it is cultured, represents a major challenge for their standardisation in vaccine formulations, potentially affecting efficacy and reproducibility.

Given these challenges, it is also necessary to state that the analyses presented here are based on studies from different research groups, and the inferences about the immunogens are based on statistical data. For this reason, certain limitations must be considered when evaluating vaccines based on ESPs and other antigens. The high heterogeneity among studies dealing with DNA vaccines, for example, may have influenced the overall effect result. It is quite challenging to generalise a result and extrapolate it to a population when there is no defined consensus on important variables in studies with immunogens against *Leishmania*. Parasite doses and infection time are some examples of important variables that are not standardised among research groups in canine leishmaniasis. Researchers do not have guidelines to rely on when conducting their studies, which hinders comparison with less heterogeneity and greater reliability among studies. To improve this condition, standardisation of research protocols with vaccines is the best way forward.

In addition, doses, routes and timing of infection may affect the reliability of the assays and potentially underestimate the efficacy of the vaccines. All experimental challenges were conducted using *L. infantum* promastigotes, a species with a well‐defined zoonotic cycle involving dogs, particularly in countries such as Brazil, where the disease is prevalent in animals and humans [3]. The parasite doses used in the experiments ranged from 10^5^–7 × 10^8^, with intravenous administration in 64.4% (29/45) of the trials. It is important to note that these challenge protocols may not accurately replicate natural infection conditions. Consequently, vaccine efficacy may be underestimated, as such a high parasite load may suppress the immune response, skewing it towards regulatory T cell (Treg) activity and a Th2‐biased response, both of which have been associated with disease progression. For example, Treg cells, particularly the IL‐10 and FoxP3^+^‐producing subsets, have been shown to contribute to immune suppression, aiding in parasite persistence in the body [39]. It is therefore possible that these cells exert immunosuppression in the face of a high parasite burden, even in vaccinated animals. Furthermore, the route of infection plays an important role. *L. infantum* is naturally transmitted by the bite of sand flies, which initiate early interactions with key immune cells such as dendritic cells (DCs), neutrophils and macrophages [41]. To address this potential bias, future studies could incorporate components of sandfly saliva into parasite challenge preparations and use doses that more closely mimic the natural route of infection [42].

To obtain sufficiently reliable information for use in this review, the studies were also subjected to risk of bias analysis using the SYRCLE's Rob Tool. This tool aims to guide authors of systematic reviews involving studies with animals and has very valid criteria that studies aiming to calculate vaccine efficacy should pay attention to [11]. However, it should be noted that this tool is designed for the evaluation of studies in mice and may not be the most suitable for assessing studies involving dogs. The issue of random spatial allocation of the animals involved in the experiment, for example, is something quite relevant to consider and is highly emphasised by the tool, as vertebrates can regulate various physiological processes through the circadian cycle, which directly interferes with their neuronal activities, leading to the production of hormones that can affect the entire body, including the functioning of the immune system [43].

Nevertheless, it is observed that 82.6% (19/23) of the studies did not contain information on the random housing of dogs, demonstrating that often these conditions, which are quite easy to apply to mice, cannot be applied in the same way to dogs due to costs, logistics and other factors. Therefore, it is necessary to emphasise that, even with uncertain or high bias for some domains of the SYRCLE tool, the studies evaluated here were considered of good quality, as much of what is evaluated by the tool is very applicable to studies with mice, but may be unfeasible for studies with dogs. This highlights the need to create a specific tool to assess the risk of bias for studies with dogs, with domains more suited to the reality of research with these animals.

## 5 Conclusions

The meta‐analysis performed in this study highlights significant differences in the efficacy of Subunit and ESP vaccines in reducing parasite load in vaccinated animals compared to unvaccinated controls. Among them, ESPs‐based vaccines demonstrated superior efficacy in parasite control, reinforcing their potential as a promising avenue for vaccine development. However, despite their strong immunogenicity, their clinical application remains uncertain due to challenges such as diagnostic interference and antigenic variability. On the other hand, DNA‐based vaccines did not show a significant impact in preventing clinical manifestations or reducing parasite load. A major limitation observed in this analysis is the high heterogeneity between studies, which makes comparisons between studies difficult. To advance the field of CVL vaccine research, it is essential to establish standardised protocols that regulate the main experimental conditions, including antigen selection, vaccine formulation, route of administration and parasite dose used for infection, as well as criteria for evaluating efficacy. This standardisation will allow more reliable comparisons between studies and provide clearer guidance for the development of effective and clinically viable vaccines against *Leishmania*.

## Author Contributions

Josiane Aparecida Martiniano de Pádua, Ricardo Toshio Fujiwara and Kelly Moura Keller conceived and designed the study. Josiane Aparecida Martiniano de Pádua, Rafaella Silva Andrade, Tuane Ferreira Melo, Marina Martins de Oliveira, Ana Laura Grossi de Oliveira and Andressa Mariana Saldanha‐Elias were responsible for data acquisition and analysis. Elaine Maria Seles Dorneles and Ana Paula Peconick contributed to data interpretation and the critical revision of the manuscript. All authors participated in drafting and revising the manuscript, approved the final version for publication, and agree to be accountable for all aspects of the work.

## Disclosure

The authors have nothing to report.

## Ethics Statement

The authors have nothing to report.

## Conflicts of Interest

The authors declare no conflicts of interest.

### Peer Review

The peer review history for this article is available at https://www.webofscience.com/api/gateway/wos/peer‐review/10.1111/pim.70006.

## Supporting information


Data S1.

Data S2.

## Data Availability

This study is a meta‐analysis and does not include primary research data. All data used in this study were obtained from previously published sources, which are cited in the manuscript. Supporting Information related to this study has been provided as part of the manuscript submission.
