# Supplementary material for: How Effective Are the Canine Visceral Leishmaniasis Vaccines Currently Being Tested in Dogs? A Systematic Review and Meta‐Analysis
Source: Parasite Immunol. 2025 Mar 3;47(3):e70006. doi: 10.1111/pim.70006 (PMC11934299; doi:10.1111/pim.70006)

**Supplementary figures**

**FIGURE S2-** Risk of bias summary: Authors' judgments of each risk of bias item for each included study, based on SYRCLE's RoB tool.


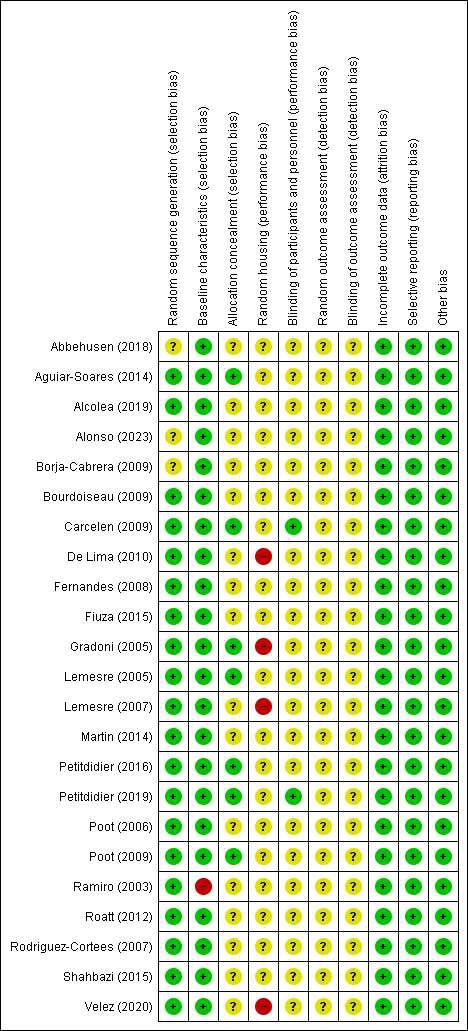


**FIGURE S3** - Risk of bias graph: Author's judgments about each risk of bias item based on in SYRCLE's RoB tool, presented as percentages across all included studies.


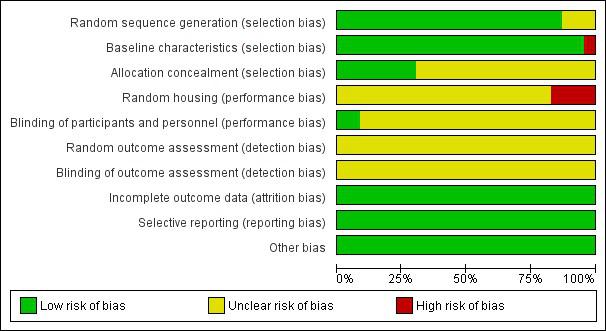

Supplement: Supplementary file 1 — Data S1. [file PIM-47-e70006-s002.docx]
