# Supplementary material for: How Effective Are the Canine Visceral Leishmaniasis Vaccines Currently Being Tested in Dogs? A Systematic Review and Meta‐Analysis
Source: Parasite Immunol. 2025 Mar 3;47(3):e70006. doi: 10.1111/pim.70006 (PMC11934299; doi:10.1111/pim.70006)
Supplement: Supplementary file 2 — Data S2. [file PIM-47-e70006-s001.docx]

**Supplementary tables**

**SUPPLEMENTARY TABLE S1** – Guidelines of PRISMA statement

| **Section/topic** | **#** | **Checklist item** | **Reported on §** |
| --- | --- | --- | --- |
| **Title** | | | |
| Title | 1 | Identify the report as a systematic review, meta-analysis, or both | §1 |
| **Abstract** | | | |
| Structured Summary | 2 | Provide s structured summary including, as applicable: background; objectives; data sources; study eligibility criteria; participants and interventions; study appraisal and synthesis methods; results; limitations; conclusions and implications of key findings; systematic review registration number | §1 |
| **Introduction** | | | |
| Rationale | 3 | Describe the rationale for the review in the context of what is already known | §1-4 |
| Objectives | 4 | Provide an explicit statement of questions being addressed with reference to participants, interventions, comparisons, outcomes, and study design (PICOS) | §4 |
| **Methods** | | | |
| Protocol and registration | 5 | Indicate if a review protocol exists, if and where it can be accessed (e.g., Web address), and, if available, provide registration information including registration number. | §1,2 |
| Eligibility criteria | 6 | Specify study characteristics (e.g., PICOS, length of follow-up) and report characteristics (e.g., years considered, language, publication status) used as criteria for eligibility, giving rationale. | §2  Tab.^1^ S2 |
| Information sources | 7 | Describe all information sources (e.g., databases with dates of coverage, contact with study authors to identify additional studies) in the search and date last searched. | §1 |
| Search | 8 | Present full electronic search strategy for at least one database, including any limits used, such that it could be repeated. | §Tab. S2 |
| Study selection | 9 | State the process for selecting studies (i.e., screening, eligibility, included in systematic review, and, if applicable, included in the meta-analysis). | §3  Tab. S3 |
| Data collection process | 10 | Describe method of data extraction from reports (e.g., piloted forms, independently, in duplicate) and any processes for obtaining and confirming data from investigators | §5-6 |
| Data items | 11 | List and define all variables for which data were sought (e.g., PICOS, funding sources) and any assumptions and simplifications made. | §4-6  Tab. S2 |
| Risk of bias in individual studies | 12 | Describe methods used for assessing risk of bias of individual studies (including specification of whether this was done at the study or outcome level), and how this information is to be used in any data synthesis | §6 |
| Summary measures | 13 | State the principal summary measures (e.g., risk ratio, difference in means). | §7 |
| Synthesis of results | 14 | Describe the methods of handling data and combining results of studies, if done, including measures of consistency (e.g., I2) for each meta-analysis. | §7 |
| Risk of bias across studies | 15 | Specify any assessment of risk of bias that may affect the cumulative evidence (e.g., publication bias, selective reporting within studies). | §6 |
| Additional analysis | 16 | Describe methods of additional analyses (e.g., sensitivity or subgroup analyses, meta-regression), if done, indicating which were pre-specified. | §7 |
| **Results** | | | |
| Study selection | 17 | Give numbers of studies screened, assessed for eligibility, and included in the review, with reasons for exclusions at each stage, ideally with a flow diagram. | §1  Fig^2^. 1 |
| Study characteristics | 18 | For each study, present characteristics for which data were extracted (e.g., study size, PICOS, follow-up period) and provide the citations. | Tab. S4 |
| Risk of bias within studies | 19 | Present data on risk of bias of each study and, if available, any outcome level assessment (see item 12). | §1  Fig.2,3 |
| Results of individual studies | 20 | For all outcomes considered (benefits or harms), present, for each study: (a) simple summary data for each intervention group (b) effect estimates and confidence intervals, ideally with a forest plot. | §2-7  Tab. 1 – 6  Tab. S5-S6 |
| Synthesis of results | 21 | Present results of each meta-analysis done, including confidence intervals and measures of consistency. | - |
| Risk of bias across studies | 22 | Present results of any assessment of risk of bias across studies (see Item 15). | - |
| Additional analysis | 23 | Give results of additional analyses, if done (e.g., sensitivity or subgroup analyses, meta-regression [see item 16)] | - |
| **Discussion** | | |  |
| Summary of evidence | 24 | Summarize the main findings including the strength of evidence for each main outcome; consider their relevance to key groups (e.g., healthcare providers, users, and policy makers) | §1 |
| Limitations | 25 | Discuss limitations at study and outcome level (e.g., risk of bias), and at review-level (e.g., incomplete retrieval of identified research, reporting bias). | §2 – 8 |
| Conclusions | 26 | Provide a general interpretation of the results in the context of other evidence, and implications for future research. | §9 |
| **Funding** | | |  |
| Funding | 27 | Describe sources of funding for the systematic review and other support (e.g., supply of data); role of funders for the systematic review. | §1 |

^1^Tab.: Table;^2^Fig.: Figure

**SUPPLEMENTARY TABLE S2 –** Search terms used in Pubmed, Web of Science, Scopus, Cochrane, Scielo and CABI, based on the PICOTS terms.

| **PICOTS** | **Search terms** |
| --- | --- |
| Population | canine OR dog* OR pupp* OR (canis AND familiaris) OR animal* |
| Intervention | AND (Leishman*) OR CanL OR CVL OR Leish-Tec OR LetiFend OR rA2 OR “recombinant A2 antigen saponin” OR Leishmune OR FML OR “Fucose Mannose ligand saponin” OR LBSap OR Leish-111f OR LeishF1 OR “recombinant *Leishmania* polyprotein LEISH-F1 antigen” OR LiESAp OR LiESA-MDP OR CaniLeish OR LACK OR “*Leishmania* Homologue of Activated C Kinase” OR “Cysteine proteinases” OR H1 OR KMP-11 OR “Kinetoplastid membrane protein-11” |
| Comparison | prophyla* OR prevalen* OR persisten* OR incidenc* OR epidemiol* OR control* OR prevent* OR efficacy OR effect* OR immun* OR protect* OR safe* OR “therapeutic use” |
| Outcomes | antibod* OR serolog* OR “clinical signs” OR alopecia OR lymphadenomegaly OR hepatosplenomegaly OR onychogryphosis OR parasit* OR “parasite load” OR xenodiagnos* |
| Time | - |
| Setting | Systematic review |

**SUPPLEMENTARY TABLE S3 –** Inclusion and exclusion criteria for selection of articles in this systematic review.

| **Inclusion criteria** | **Exclusion criteria** |
| --- | --- |
| - All countries | - *In vitro* and *In silico* studies |
| - All years | - Cohort studies |
| - Canine visceral leishmaniasis | - Case-control studies |
| - Studies on vaccines efficacy | - Cross-sectional studies |
| - *L. infantum* | - Case reports and Reviews |
| - Vaccines tested on dogs | - Diagnostic performance of tests |
|  | - Therapeutics |
|  | - Languages other than English, Spanish or Portuguese |
|  | - Full-text not available |
|  | - No information about control group |
|  | - No information about vaccine antigen, dose, route, number of vaccinations or time between doses |
|  | - No challenge performed |
|  | - No information about challenge dose, route, strain or time |
|  | - No information about evaluation of humoral immune response |
|  | - No information about evaluation of cellular immune response |
|  | - No information about parasite load |
|  | - No information about vaccine protection rate |
|  |  |

**SUPPLEMENTARY TABLE S4** – Studies not selected by quality criteria in this review

| **First author, year** | **Reason** |
| --- | --- |
| Araújo, 2011 | No challenge |
| Araújo, 2009 | No challenge |
| Bongiorno, 2013 | Humoral or cellular immune response not evaluated |
| Borja-Cabrera,2012 | Humoral or cellular immune response not evaluated |
| Borja-Cabrera, 2008 | No control group |
| Borja-Cabrera, 2010 | Humoral or cellular immune response not evaluated |
| Borja-Cabrera, 2002 | Humoral or cellular immune response not evaluated |
| Cacheiro-Laguno, 2020 | Humoral or cellular immune response not evaluated |
| Carillo, 2008 | Humoral or cellular immune response not evaluated |
| Cotrina, 2018 | Humoral or cellular immune response not evaluated |
| Daneshvar, 2010 | No vaccine information |
| de Amorim, 2010 | No challenge |
| Dunan, 1989 | Humoral or cellular immune response not evaluated |
| Fallah, 1998 | Humoral or cellular immune response not evaluated |
| Fernandes, 2014 | Humoral or cellular immune response not evaluated |
| Fujiwara, 2005 | Humoral or cellular immune response not evaluated |
| Giunchetti, 2007 | No challenge |
| Giunchetti, 2008 | No challenge |
| Giunchetti, 2008 | No challenge |
| Grimaldi, 2017 | Humoral or cellular immune response not evaluated |
| Vitoriano-Souza, 2008 | Humoral or cellular immune response not evaluated |
| Lopes, 2018 | Humoral or cellular immune response not evaluated |
| Martinez-Rodrigo, 2019 | No challenge |
| Moreira, 2016 | No challenge |
| Moreno, 2007 | No challenge |
| Moreno, 2013 | No challenge |
| Moreno, 2014 | No challenge |
| Nogueira, 2005 | Humoral or cellular immune response not evaluated |
| Oliva, 2014 | Humoral or cellular immune response not evaluated |
| Silva, 2016 | Humoral or cellular immune response not evaluated |
| Resende, 2016 | Humoral or cellular immune response not evaluated |
| Resende, 2013 | Humoral or cellular immune response not evaluated |
| Saraiva, 2006 | Humoral or cellular immune response not evaluated |
| Testasicca, 2014 | Humoral or cellular immune response not evaluated |
| Vannucci, 2018 | No challenge |
| Velez, 2020 | No challenge |
| Souza, 2013 | No challenge |
| Zarei, 2023 | No challenge |
| Ogunkolade, 1988 | No challenge |

**SUPPLEMENTARY TABLE S5 –** Vaccination and challenge data from trials selected for systematic review and meta-analysis

| **First author, year** |  | **N Vac^c^** | **N C^d^** |  | **Vaccination** | | | **Challenge** | | | |
| --- | --- | --- | --- | --- | --- | --- | --- | --- | --- | --- | --- |
|  | **Vaccine’s generation** |  |  | **Control Group** | **Antigen/Adjuvant** | **Age (Months)** | **Route** | **Type of Challenge** | **Strain** | **Dose^s^** | **Route** |
| Abbehusen, 2018^u^ | 3a^a^ | 10 | 10 | P^x^ | LJM17 | 2-3 | IM^m^ | E^o^ | LIP^q^ | 10^7^ | ID^l^ |
| Abbehusen, 2018^u^ | 3a^a^ | 10 | 10 | P^x^ | LJL143 | 2-3 | IM^m^ | E^o^ | LIP^q^ | 10^7^ | ID^l^ |
| Aguiar-Soares, 2014 | 2a^b^ | 5 | 5 | P^x^ | LB^f^ + SGE^g^/Saponin | 7- 8 | SC^k^ | E^o^ | LIP^q^ | 10^7^ | ID^l^ |
| Aguiar-Soares, 2014 | 2a^b^ | 5 | 5 | P^x^ | LB^f^ + SGE^g^ | 7-8 | SC^k^ | E^o^ | LIP^q^ | 10^7^ | ID^l^ |
| Aguiar-Soares, 2014 | 2a^b^ | 5 | 5 | P^x^ | SGE^g^ | 7-8 | SC^k^ | E^o^ | LIP^q^ | 10^7^ | ID^l^ |
| Alcolea, 2019 | 3a^a^ | 5 | 5 | P^x^ | pPAL-LACK | 12-18 | IN^n^ | E^o^ | LIP^q^ | 10^8^ | UN^e^ |
| Borja-Cabrera, 2009 | 3a^a^ | 6 | 13 | P^x^ | VR1012-NH36 | 4 | IM^m^ | E^o^ | LIA^r^ | 7x10^8^ | UN^e^ |
| Bourdoiseau, 2009^v^ | 2a^b^ | 3 | 3 | A^y^ | LiESAp/MDP | UN^e^ | SC^k^ | E^o^ | LIP^q^ | 10^8^ | ID^l^ |
| Bourdoiseau, 2009^v^ | 2a^b^ | 3 | 3 | A^y^ | LiESAp/MDP | UN^e^ | SC^k^ | E^o^ | LIP^q^ | 10^8^ | ID^l^ |
| Carcelen, 2009 | 2a^b^ | 7 | 7 | P^x^ | Q-Protein | 12-24 | SC^k^ | E^o^ | LIP^q^ | 10^5^ | IV^t^ |
| Carcelen, 2009 | 2a^b^ | 7 | 7 | P^x^ | Q-Protein | 12-24 | SC^k^ | E^o^ | LIP^q^ | 10^5^ | IV^t^ |
| De Lima, 2010 | 2a^b^ | 20 | 20 | P^x^ | FML | UN^e^ | SC^k^ | NC^p^ | NC^p^ | NC^p^ | NC^p^ |
| De Lima, 2010 | 2a^b^ | 20 | 20 | P^x^ | FML | UN^e^ | SC^k^ | NC^p^ | NC^p^ | NC^p^ | NC^p^ |
| Fernandes, 2008 | 2a^b^ | 14 | 7 | P^x^ | rA2/Saponin | 3-9 | SC^k^ | E^o^ | LIP^q^ | 5x10^7^ | IV^t^ |
| Fernandes, 2008 | 2a^b^ | 14 | 7 | P^x^ | rA2/Saponin | 3-9 | SC^k^ | E^o^ | LIP^q^ | 5x10^7^ | IV^t^ |
| Fernandes, 2008 | 2a^b^ | 14 | 4 | P^x^ | rA2/Saponin | 3-9 | SC^k^ | E^o^ | LIP^q^ | 5x10^7^ | IV^t^ |
| Fernandes, 2008 | 2a^b^ | 14 | 3 | A^y^ | rA2/Saponin | 3-9 | SC^k^ | E^o^ | LIP^q^ | 5x10^7^ | IV^t^ |
| Fiuza, 2015 | 2a^b^ | 6 | 6 | P^x^ | LdCen | 8 | SC^k^ | E^o^ | LIP^q^ | 10^7^ | IV^t^ |
| Fiuza, 2015 | 2a^b^ | 6 | 6 | P^x^ | FML | 8 | SC^k^ | E^o^ | LIP^q^ | 10^7^ | IV^t^ |
| Gradoni, 2005 | 2a^b^ | 15 | 15 | P^x^ | MML/ MPL-SE | 6 | SC^k^ | NC^p^ | NC^p^ | NC^p^ | NC^p^ |
| Gradoni, 2005 | 2a^b^ | 15 | 15 | P^x^ | MML/ Adjuprime | 6 | SC^k^ | NC^p^ | NC^p^ | NC^p^ | NC^p^ |
| Lemesre, 2005 | 2a^b^ | 3 | 3 | A^y^ | LiESAp/MDP | 12-72 | SC^k^ | E^o^ | LIP^q^ | 10^8^ | IV^t^ |
| Lemesre, 2005 | 2a^b^ | 3 | 3 | A^y^ | LiESAp/MDP | 12-72 | SC^k^ | E^o^ | LIP^q^ | 10^8^ | IV^t^ |
| Lemesre, 2005 | 2a^b^ | 3 | 3 | A^y^ | LiESAp/MDP | 12-72 | SC^k^ | E^o^ | LIP^q^ | 10^8^ | IV^t^ |
| Lemesre, 2005 | 2a^b^ | 3 | 3 | A^y^ | LiESAp/MDP | 12-72 | SC^k^ | E^o^ | LIP^q^ | 10^8^ | IV^t^ |
| Lemesre, 2007 | 2a^b^ | UN^e^ | UN^e^ | P^x^ | LiESAp/MDP | UN^e^ | SC^k^ | NC^p^ | NC^p^ | NC^p^ | NC^p^ |
| Martin, 2014 | 2a^b^ | 10 | 10 | P^x^ | LiESP/QA-21 | 6 | SC^k^ | E^o^ | LIP^q^ | 10^8,5^ | IV^t^ |
| Petitdidier, 2016 | 2a^b^ | 9 | 5 | P^x^ | LaPSA-38S/QA-21 | 24-48 | SC^k^ | E^o^ | LIP^q^ | 10^8^ | IV^t^ |
| Petitdidier, 2016 | 2a^b^ | 5 | 5 | P^x^ | LaPSA-12S/QA-21 | 24-48 | SC^k^ | E^o^ | LIP^q^ | 10^8^ | IV^t^ |
| Petitdidier, 2019 | 2a^b^ | 10 | 5 | P^x^ | A17G + A17E + E34PC/QA-21 | 24-48 | SC^k^ | E^o^ | LIP^q^ | 10^8^ | IV^t^ |
| Poot, 2009 | 2a^b^ | 7 | 7 | P^x^ | rJPCM5_Q^h^/ MDP | 6 | SC^k^ | E^o^ | LIP^q^ | 5x10^7^ | IV^t^ |
| Poot, 2009 | 2a^b^ | 7 | 7 | P^x^ | rJPCM5_Q^h^/ Aluminum hydroxide | 6 | SC^k^ | E^o^ | LIP^q^ | 5x10^7^ | IV^t^ |
| Poot, 2009 | 2a^b^ | 7 | 7 | P^x^ | rJPCM5_Q^h^/ ISCOMatrix C | 6 | SC^k^ | E^o^ | LIP^q^ | 5x10^7^ | IV^t^ |
| Poot, 2009 | 2a^b^ | 5 | 1 | P^x^ | rJPCM5_Q^i^/ MDP | 6 | SC^k^ | E^o^ | LIP^q^ | 5x10^7^ | IV^t^ |
| Poot, 2009 | 2a^b^ | 5 | 1 | P^x^ | rJPCM5_Q^i^/ Aluminum hydroxide | 6 | SC^k^ | E^o^ | LIP^q^ | 5x10^7^ | IV^t^ |
| Poot, 2009 | 2a^b^ | 5 | 1 | P^x^ | rJPCM5_Q^i^/ ISCOMatrix C | 6 | SC^k^ | E^o^ | LIP^q^ | 5x10^7^ | IV^t^ |
| Poot, 2006 | 2a^b^ | 5 | 5 | P^x^ | rCPA + rCPB/ rIL-12 | 6 | SC^k^ | E^o^ | LIP^q^ | 5x10^7^ | IV^t^ |
| Poot, 2006 | 2a^b^ | 5 | 5 | P^x^ | rCPA + rCPB/ rIL-12 + QuilA | 6 | SC^k^ | E^o^ | LIP^q^ | 5x10^7^ | IV^t^ |
| Ramiro, 2003 | 3a^a^ | 5 | 5 | P^x^ | DNA-LACK | 18-54 | SC^k^ | E^o^ | LIP^q^ | 10^8^ | IV^t^ |
| Ramiro, 2003 | 3a^a^ | 5 | 5 | P^x^ | DNA-LACK + rVV-LACK | 18-54 | SC^k^ | E^o^ | LIP^q^ | 10^8^ | IV^t^ |
| Roatt, 2012 | 2a^b^ | 5 | 5 | P^x^ | LBf/Saponin | UN^e^ | SC^k^ | E^o^ | LIP^q^ | 10^7^ | ID^l^ |
| Rodriguez-Cortés, 2007 | 3a^a^ | 6 | 6 | P^x^ | pMOK-Kmp11/-TRYP/-LACK/-GP63 | 9 | ID^l^ | E^o^ | LIP^q^ | 5x10^7^ | IV^t^ |
| Shahbazi, 2015 | 3a^a^ | 10 | 10 | P^x^ | pcDNA-A2-CPACPB−CTEGF P (cSLN) | 6-48 | SC^k^ | E^o^ | LIP^q^ | 4x10^7^ | IV^t^ |
| Shahbazi, 2015 | 3a^a^ | 10 | 10 | P^x^ | pcDNA-A2-CPACPB−CTEGFP (Electroporation) | 6-48 | SC^k^ | E^o^ | LIP^q^ | 4x10^7^ | IV^t^ |
| Velez, 2020^w^ | 2a^b^ | 85 | 83 | P^x^ | LiESP/Saponin | >6^j^ | SC^k^ | NC^p^ | NC^p^ | NC^p^ | NC^p^ |

^a^3a: Third Generation; ^b^2a: Second Generation; ^c^N Vac: number of vaccinated animals; ^d^N C: number of control animals; ^e^UN: Uninformed; ^f^LB: *L. braziliensis* protein; ^g^SGE: Sand fly salivary gland extract*;* ^h^rJPCM5_Q: Antigen produced by *E. coli;* ^i^rJPCM5_Q: Antigen produced by *Baculovirus;* ^j^>6: More than 6 months; ^k^SC: subcutaneous; ^l^ID: intradermal, ^m^IM: intramuscular; ^n^IN: Intranasal; ^o^E: Experimental challenge; ^p^NC: Natural challenge; ^q^LIP: *L. infantum* promastigotes; ^r^LIA: *L. infantum* amastigotes; ^s^Dose: Unity = Parasites; ^t^IV: Intravenous. ^u^This trial has been corrected and its errata were also considered in this review. ^v^Only some data were taken from this trial, which fit the quality criteria. ^w^In this article, the number of animals considered were the ones that ended up as vaccinations and did all the tests; ^x^P: Placebo; ^y^A: Adjuvant

**SUPPLEMENTARY TABLE S6** – Detailed information about the vaccinations in the trials that performed this analysis among those selected by this systematic review and meta-analysis.

|  |  |  | **First vaccination** | | **Second vaccination** | | | **Third vaccination** | | | **Fourth vaccination** | | |
| --- | --- | --- | --- | --- | --- | --- | --- | --- | --- | --- | --- | --- | --- |
| **First author, year** | **Route** | **Age** | **Antigen** | **Dose** | **Antigen** | **Interval^a^** | **Dose** | **Antigen** | **Interval^a^** | **Dose** | **Antigen** | **Interval^a^** | **Dose** |
| Abbehusen, 2018 | IM^o^ | 2-3 m^g^ | LJM17^i^ | 250 µg | LJM17^x^ | 28 d^j^ | 10^8 | LJM17^x^ | 42 d^j^ | 10^8 | NP^l^ | NP^l^ | NP^l^ |
| Abbehusen, 2018 | IM^o^ | 2-3 m^g^ | LJL143^i^ | 250 µg | LJL143^x^ | 28 d^j^ | 10^8 | LJL143^x^ | 42 d^j^ | 10^8 | NP^l^ | NP^l^ | NP^l^ |
| Aguiar-Soares, 2014 | SC^m^ | 7- 8 m^g^ | LB^b^ + SGE^c^ | 600µg^q^ | Same^y^ | 28 d^j^ | Same^y^ | Same^y^ | 28 d^j^ | Same^y^ | NP^l^ | NP^l^ | NP^l^ |
| Aguiar-Soares, 2014 | SC^m^ | 7-8 m^g^ | LB^b^ + SGE^c^ | 600µg^q^ | Same^y^ | 28 d^j^ | Same^y^ | Same^y^ | 28 d^j^ | Same^y^ | NP^l^ | NP^l^ | NP^l^ |
| Aguiar-Soares, 2014 | SC^m^ | 7-8 m^g^ | SGE^c^ | UN^f^ | Same^y^ | 28 d^j^ | Same^y^ | Same^y^ | 28 d^j^ | Same^y^ | NP^l^ | NP^l^ | NP^l^ |
| Alcolea, 2019 | IN^p^ | 12-18 m^g^ | pPAL-LACK + pPAL-canIL12-p35 + pPALcanIL12-p40 | 200μg^r^ + 20μg^s^ + 20μg^s^ | Same^y^ | 15 d^j^ | Same^y^ | Same^y^ | 15 d^j^ | Same^y^ | NP^l^ | NP^l^ | NP^l^ |
| Borja-Cabrera, 2009 | IM^o^ | 4 m^g^ | VR1012-NH36 | 750 μg | Same^y^ | 21 d^j^ | Same^y^ | Same^y^ | 21 d^j^ | Same^y^ | NP^l^ | NP^l^ | NP^l^ |
| Bourdoiseau, 2009 | SC^m^ | UN^f^ | LiESAp | 100 μg | Same^y^ | 21 d^j^ | Same^y^ | Same^y^ | 21 d^j^ | Same^y^ | NP^l^ | NP^l^ | NP^l^ |
| Bourdoiseau, 2009 | SC^m^ | UN^f^ | LiESAp | 100 μg | Same^y^ | 21 d^j^ | Same^y^ | Same^y^ | 21 d^j^ | Same^y^ | NP^l^ | NP^l^ | NP^l^ |
| Carcelen, 2009 | SC^m^ | 12-24 m^g^ | Q protein | 100 μg | NP^l^ | NP^l^ | NP^l^ | NP^l^ | NP^l^ | NP^l^ | NP^l^ | NP^l^ | NP^l^ |
| Carcelen, 2009 | SC^m^ | 12-24 m^g^ | Q protein | 100 μg | Same^y^ | 21 d^j^ | Same^y^ | NP^l^ | NP^l^ | NP^l^ | NP^l^ | NP^l^ | NP^l^ |
| De Lima, 2010 | SC^m^ | UN^f^ | FML | 1 mL^t^ | Same^y^ | 21 d^j^ | Same^y^ | Same^y^ | 21 d^j^ | Same^y^ | NP^l^ | NP^l^ | NP^l^ |
| De Lima, 2010 | SC^m^ | UN^f^ | FML | 1mL^t^ | Same^y^ | 21 d^j^ | Same^y^ | Same^y^ | 21 d^j^ | Same^y^ | NP^l^ | NP^l^ | NP^l^ |
| Fernandes, 2008 | SC^m^ | 3-9 m^g^ | rA2 | 100 μg^u^ | Same^y^ | 21 d^j^ | Same^y^ | Same^y^ | 21 d^j^ | Same^y^ | NP^l^ | NP^l^ | NP^l^ |
| Fernandes, 2008 | SC^m^ | 3-9 m^g^ | rA2 | 100 μg | Same^y^ | 21 d^j^ | Same^y^ | Same^y^ | 21 d^j^ | Same^y^ | NP^l^ | NP^l^ | NP^l^ |
| Fernandes, 2008 | SC^m^ | 3-9 m^g^ | rA2 | 100 μg | Same^y^ | 21 d^j^ | Same^y^ | Same^y^ | 21 d^j^ | Same^y^ | NP^l^ | NP^l^ | NP^l^ |
| Fernandes, 2008 | SC^m^ | 3-9 m^g^ | rA2 | 100 μg | Same^y^ | 21 d^j^ | Same^y^ | Same^y^ | 21 d^j^ | Same^y^ | NP^l^ | NP^l^ | NP^l^ |
| Fiuza, 2015 | SC^m^ | 8 m^g^ | FML | 1mL^t^ | Same^y^ | 21 d^j^ | Same^y^ | Same^y^ | 21 d^j^ | Same^y^ | NP^l^ | NP^l^ | NP^l^ |
| Fiuza, 2015 | SC^m^ | 8 m^g^ | LdCen | 10^7 | NP^l^ | NP^l^ | NP^l^ | NP^l^ | NP^l^ | NP^l^ | NP^l^ | NP^l^ | NP^l^ |
| Gradoni, 2005 | SC^m^ | 6 m^g^ | MML | 45μg | Same^y^ | 21 d^j^ | Same^y^ | Same^y^ | 21 d^j^ | Same^y^ | NP^l^ | NP^l^ | NP^l^ |
| Gradoni, 2005 | SC^m^ | 6 mg | MML | 45μg | Same^y^ | 28 d^j^ | Same^y^ | Same^y^ | 21 d^j^ | Same^y^ | NP^l^ | NP^l^ | NP^l^ |
| Lemesre, 2005 | SC^m^ | 12-72 m^g^ | LiESAp | 50μg | Same^y^ | 28 d^j^ | Same^y^ | NP^l^ | NP^l^ | NP^l^ | NP^l^ | NP^l^ | NP^l^ |
| Lemesre, 2005 | SC^m^ | 12-72 m^g^ | LiESAp | 100 μg | Same^y^ | 21 d^j^ | Same^y^ | NP^l^ | NP^l^ | NP^l^ | NP^l^ | NP^l^ | NP^l^ |
| Lemesre, 2005 | SC^m^ | 12-72 m^g^ | LiESAp | 100 μg | Same^y^ | 21 d^j^ | Same^y^ | NP^l^ | NP^l^ | NP^l^ | NP^l^ | NP^l^ | NP^l^ |
| Lemesre, 2005 | SC^m^ | 12-72 m^g^ | LiESAp | 200 μg | Same^y^ | 21 d^j^ | Same^y^ | NP^l^ | NP^l^ | NP^l^ | NP^l^ | NP^l^ | NP^l^ |
| Lemesre, 2007 | SC^m^ | UN^f^ | LiESAp | 100μg | Same^y^ | 3-4 w^k^ | Same^y^ | Same^y^ | 3-4 w^k^ | Same^y^ | NP^l^ | NP^l^ | NP^l^ |
| Martin, 2014 | SC^m^ | 6 m^g^ | LiESP/QA-21 | 100 μg | Same^y^ | 21 d^j^ | Same^y^ | Same^y^ | 21 d^j^ | Same^y^ | NP^l^ | NP^l^ | NP^l^ |
| Petitdidier, 2016 | SC^m^ | 24-48 m^g^ | LaPSA-38S | 25 μg  25 μg | Same^y^ | 28 d^j^ | Same^y^ | Same^y^ | 28 d^j^ | Same^y^ | NP^l^ | NP^l^ | NP^l^ |
| Petitdidier, 2016 | SC^m^ | 24-48 m^g^ | LaPSA-12S | 25 μg | Same^y^ | 28 d^j^ | Same^y^ | Same^y^ | 28 d^j^ | Same^y^ | NP^l^ | NP^l^ | NP^l^ |
| Petitdidier, 2019 | SC^m^ | 24-48 m^g^ | A17G + A17E + E34PC | 25 μg^v^ + 25 μg^v^ + 10 μg^w^ | Same^y^ | 28 d^j^ | Same^y^ | Same^y^ | 28 d^j^ | Same^y^ | NP^l^ | NP^l^ | NP^l^ |
| Poot, 2009 | SC^m^ | 6 m^g^ | rJPCM5_Q^d^ | 70μg | Same^y^ | 21 d^j^ | Same^y^ | NP^l^ | NP^l^ | NP^l^ | NP^l^ | NP^l^ | NP^l^ |
| Poot, 2009 | SC^m^ | 6 m^g^ | rJPCM5_Q^d^ | 70μg | Same^y^ | 21 d^j^ | Same^y^ | NP^l^ | NP^l^ | NP^l^ | NP^l^ | NP^l^ | NP^l^ |
| Poot, 2009 | SC^m^ | 6 m^g^ | rJPCM5_Q^d^ | 70μg | Same^y^ | 21 d^j^ | Same^y^ | NP^l^ | NP^l^ | NP^l^ | NP^l^ | NP^l^ | NP^l^ |
| Poot, 2009 | SC^m^ | 6 m^g^ | rJPCM5_Q^e^ | 70μg | Same^y^ | 21 d^j^ | Same^y^ | NP^l^ | NP^l^ | NP^l^ | NP^l^ | NP^l^ | NP^l^ |
| Poot, 2009 | SC^m^ | 6 m^g^ | rJPCM5_Q^e^ | 70μg | Same^y^ | 21 d^j^ | Same^y^ | NP^l^ | NP^l^ | NP^l^ | NP^l^ | NP^l^ | NP^l^ |
| Poot, 2009 | SC^m^ | 6 m^g^ | rJPCM5_Q^e^ | 70μg | Same^y^ | 21 d^j^ | Same^y^ | NP^l^ | NP^l^ | NP^l^ | NP^l^ | NP^l^ | NP^l^ |
| Poot, 2006 | SC^m^ | 6 m^g^ | rCPA + rCPB | 50 μg + 50 μg | Same^y^ | 28 d^j^ | Same^y^ | NP^l^ | NP^l^ | NP^l^ | NP^l^ | NP^l^ | NP^l^ |
| Poot, 2006 | SC^m^ | 6 m^g^ | rCPA + rCPB | 50 μg + 50 μg | Same^y^ | 28 d^j^ | Same^y^ | NP^l^ | NP^l^ | NP^l^ | NP^l^ | NP^l^ | NP^l^ |
| Ramiro, 2003 | SC^m^ | 18-54 m^g^ | DNA-LACK | 100µg | Same^y^ | 15 d^j^ | Same^y^ | NP^l^ | NP^l^ | NP^l^ | NP^l^ | NP^l^ | NP^l^ |
| Ramiro, 2003 | SC^m^ | 18-54 m^g^ | DNA-LACK | 100µg | rVV-LACK | 15 d^j^ | 10^8 pfu | NP^l^ | NP^l^ | NP^l^ | NP^l^ | NP^l^ | NP^l^ |
| Roatt, 2012 | SC^m^ | UN^f^ | LB^b^ | 600µg | Same^y^ | 28 d^j^ | Same^y^ | Same^y^ | 21 d^j^ | Same^y^ | NP^l^ | NP^l^ | NP^l^ |
| Roatt, 2012 | SC^m^ | UN^f^ | LB^b^ | 600µg | Same^y^ | 28 d^j^ | Same^y^ | Same^y^ | 21 d^j^ | Same^y^ | NP^l^ | NP^l^ | NP^l^ |
| Roatt, 2012 | SC^m^ | UN^f^ | Saponin | 600µg | Same^y^ | 28 d^j^ | Same^y^ | Same^y^ | 21 d^j^ | Same^y^ | NP^l^ | NP^l^ | NP^l^ |
| Rodriguez-Cortés, 2007 | ID^n^ | 9 m^g^ | pMOK-Kmp11/-TRYP/-LACK/-GP63 | 200µg^w^ | Same^y^ | 15 d^j^ | Same^y^ | Same^y^ | 15 d^j^ | Same^y^ | Same^y^ | Same^y^ | 15 d^j^ |
| Shahbazi, 2015 | SC^m^ | 6-48 m^g^ | pcDNA-A2-CPACPB−CTEGF P (cSLN) | 200µg | Same^y^ | 21 d^j^ | Same^y^ | NP^l^ | NP^l^ | NP^l^ | NP^l^ | NP^l^ | NP^l^ |
| Shahbazi, 2015 | SC^m^ | 6-48 m^g^ | pcDNA-A2-CPACPB−CTEGFP (Electroporation) | 200µg | Same^y^ | 21 d^j^ | Same^y^ | NP^l^ | NP^l^ | NP^l^ | NP^l^ | NP^l^ | NP^l^ |
| Velez, 2020 | SC^m^ | >6^h^ m^g^ | LiESP | 100 μg | Same^y^ | 21 d^j^ | Same^y^ | Same^y^ | 21 d^j^ | Same^y^ | NP^l^ | NP^l^ | NP^l^ |

^a^Interval: interval between the vaccinations; ^b^LB: *L. braziliensis* protein; ^c^SGE: Sand fly salivary gland extract*;* ^d^rJPCM5_Q: Antigen produced by *E. coli;* ^e^rJPCM5_Q: Antigen produced by *Baculovirus;* ^f^UN: Uninformed; ^g^m: Months; ^h^>6: More than 6 months; ^i^DNA plasmid; ^j^d: Days; ^k^w: Weeks; ^l^NP: Not performed; ^m^SC: subcutaneous; ^n^ID: intradermal, ^o^IM: intramuscular; ^p^IN: Intranasal; ^q^Dose of LB protein; ^r^Dose of pPAL-LACK; ^s^Doses of pPAL-canIL12-p35 and pPALcanIL12-p40; ^t^Dose recommended by the vaccine manufacturer; ^u^Doses of A17G and A17E; ^v^Dose of E34PC; ^w^Dose of each plasmid; ^x^Expressed by *Canarypoxvirus;* ^y^Same antigen and dose as first dose; ^x^Promastigotes of *L. infantum*

**SUPPLEMENTARY TABLE S7 –** Detailed data on the humoral immune response evaluated through antibodies against the vaccine antigen, produced after vaccination, in trails that performed an experimental challenge and performed this analysis among those selected by this systematic review.

| **First author, year** | **Antigen/Adjuvant** | **ELISA (Antibodies)^a^** | | **Cut off (ELISA)** | **IFAT^b^** | |
| --- | --- | --- | --- | --- | --- | --- |
|  |  | **Nvac^c^ (%)** | **NC^d^ (%)** |  | **Nvac^c^ (%)** | **NC^d^ (%)** |
| Abbehusen, 2018 | LJM17 | 10/10 (100) | 0 | 0.200 | NP^e^ | NP^e^ |
| Abbehusen, 2018 | LJL143 | 10/10 (100) | 0 | 0.195 | NP^e^ | NP^e^ |
| Aguiar-Soares, 2014 | LB + SGE/Saponin | UE^f^ | UE^f^ | UE^f^ | NP^e^ | NP^e^ |
| Aguiar-Soares, 2014 | LB + SGE | UE^f^ | UE^f^ | UE^f^ | NP^e^ | NP^e^ |
| Aguiar-Soares, 2014 | SGE | UE^f^ | UE^f^ | UE^f^ | NP^e^ | NP^e^ |
| Alcolea, 2019 | pPAL-LACK | NP^e^ | NP^e^ | NP^e^ | NP^e^ | NP^e^ |
| Borja-Cabrera, 2009 | VR1012-NH36 | UE^f^ | UE^f^ | UE^f^ | NP^e^ | NP^e^ |
| Bourdoiseau, 2009 | LiESAp/MDP | UE^f^ | UE^f^ | UE^f^ | NP^e^ | NP^e^ |
| Bourdoiseau, 2009 | LiESAp/MDP | UE^f^ | UE^f^ | UE^f^ | NP^e^ | NP^e^ |
| Carcelen, 2009 | Q-Protein | UE^f^ | UE^f^ | UE^f^ | NP^e^ | NP^e^ |
| Carcelen, 2009 | Q-Protein | UE^f^ | UE^f^ | UE^f^ | NP^e^ | NP^e^ |
| Fernandes, 2008 | rA2/Saponin | UE^f^ | UE^f^ | UE^f^ | NP^e^ | NP^e^ |
| Fiuza, 2015 | LdCen | UE^f^ | UE^f^ | UE^f^ | NP^e^ | NP^e^ |
| Fiuza, 2015 | FML | UE^f^ | UE^f^ | UE^f^ | NP^e^ | NP^e^ |
| Lemesre, 2005 | LiESAp/MDP | UE^f^ | UE^f^ | UE^f^ | NP^e^ | NP^e^ |
| Lemesre, 2005 | LiESAp/MDP | UE^f^ | UE^f^ | UE^f^ | NP^e^ | NP^e^ |
| Lemesre, 2005 | LiESAp/MDP | UE^f^ | UE^f^ | UE^f^ | NP^e^ | NP^e^ |
| Lemesre, 2005 | LiESAp/MDP | UE^f^ | UE^f^ | UE^f^ | NP^e^ | NP^e^ |
| Martin, 2014 | LiESP/QA-2/QA-21 | 7/10 (70) | 0 | UE^f^ | NP^e^ | NP^e^ |
| Petitdidier, 2016 | LaPSA-38S/QA-21 | UE^f^ | UE^f^ | UE^f^ | NP^e^ | NP^e^ |
| Petitdidier, 2016 | LaPSA-12S/QA-21 | UE^f^ | UE^f^ | UE^f^ | NP^e^ | NP^e^ |
| Petitdidier, 2019 | A17G + A17E + E34PC/QA-21 | UE^f^ | UE^f^ | UE^f^ | NP^e^ | NP^e^ |
| Poot, 2009 | rJPCM5_Q/MDP | UE^f^ | UE^f^ | UE^f^ | NP^e^ | NP^e^ |
| Poot, 2009 | rJPCM5_Q/ Aluminum hydroxide | UE^f^ | UE^f^ | UE^f^ | NP^e^ | NP^e^ |
| Poot, 2009 | rJPCM5_Q/  ISCOMatrix C | UE^f^ | UE^f^ | UE^f^ | NP^e^ | NP^e^ |
| Poot, 2009 | rJPCM5_Q/MDP | UE^f^ | UE^f^ | UE^f^ | NP^e^ | NP^e^ |
| Poot, 2006 | rCPA + rCPB | UE^f^ | UE^f^ | UE^f^ | NP^e^ | NP^e^ |
| Ramiro, 2003 | DNA-LACK | UE^f^ | UE^f^ | UE^f^ | NP^e^ | NP^e^ |
| Ramiro, 2003 | DNA-LACK + rVV-LACK | UE^f^ | UE^f^ | UE^f^ | NP^e^ | NP^e^ |
| Roatt, 2012 | LBf/ Saponin | NP^e^ | NP^e^ | NP^e^ | NP^e^ | NP^e^ |
| Rodriguez-Cortees, 2007 | pMOK-Kmp11/-TRYP/-LACK/-GP63 | 6/6 (100) | 2/6 (33.33) | 9 EU | NP^e^ | NP^e^ |
| Shahbazi, 2015 | pcDNA-A2-CPACPB−CTEGFP (cSLN) | UE^f^ | UE^f^ | UE^f^ | NP^e^ | NP^e^ |
| Shahbazi, 2015 | pcDNA-A2-CPACPB−CTEGFP (Electroporation) | UE^f^ | UE^f^ | UE^f^ | NP^e^ | NP^e^ |

^a^Number of animals that developed a humoral immune response against the vaccine antigen confirmed by ELISA; ^b^Number of animals that developed a humoral immune response against the vaccine antigen confirmed by IFAT; cNVac: Vaccinated group; ^d^NC: Control group; ^e^NP: Not performed; ^f^UE: Unable exctract;

**SUPPLEMENTARY TABLE S8 –** Detailed data on the humoral immune response evaluated through antibodies against the vaccine antigen, produced after vaccination, in trails that performed an natural challenge and performed this analysis among those selected by this systematic review

| **First author, year** | **Antigen/Adjuvant** | **ELISA (Antibodies)^a^** | |  | **IFAT^b^** | |
| --- | --- | --- | --- | --- | --- | --- |
|  |  | **Nvac^c^**  **(%)** | **NC^d^**  **(%)** | **Cut off (ELISA)** | **Nvac^c^**  **(%)** | **NC^d^**  **(%)** |
| De Lima, 2010 | FML | UE^g^ | UE^g^ | UE^g^ | NP^e^ | NP^e^ |
| Gradoni, 2005^f^ | MML + MPL-SE | 15/15 (100) | 1/15 (6.67) | UE^g^ | NP^e^ | NP^e^ |
| Gradoni, 2005 ^f^ | MML + Adjuprime | 8/15 (53.33) | 1/15 (6.67) | UE^g^ | NP^e^ | NP^e^ |
| Lemesre, 2007 | LiESAp + MDP | 21/22 (98.2) | 1/33 (2.3) | 0.128 | NP^e^ | NP^e^ |
| Velez, 2020 | LiESP + Saponina | NP^e^ | NP^e^ | NP^e^ | NP^e^ | NP^e^ |

^a^Number of animals that developed a humoral immune response against the vaccine antigen confirmed by ELISA; ^b^Number of animals that developed a humoral immune response against the vaccine antigen confirmed by IFAT; ^c^Vaccinated group; ^d^Control group; ^e^NP: Not Performed; ^f^ Results of tests performed 1 year after vaccination; ^g^UE: Unable extract;
